# Wearable device-measured physical activity and incident cardiovascular disease in cancer survivors

**DOI:** 10.1136/bjsports-2024-108734

**Published:** 2025-03-11

**Authors:** Chengqing Jiang, Ziang Li, Bo Guo, Lin Chen, Liang Zhu, Yu Liang, Yinghan Shen, Tianxin Long, Ming Zhai, Jiayun Shi, Haiyan Xu, Yongjian Wu

**Affiliations:** 1 Department of Cardiology, Fuwai Hospital, National Center for Cardiovascular Disease, Chinese Academy of Medical Science and Peking Union Medical College, Beijing, China; 2 Department of Medicine for Sports and Performing Arts, Osaka University Graduate School of Medicine, 2-2 Yamadaoka, Suita, Osaka, Japan; 3 Department of Radiation Oncology, State Key Laboratory of Oncology in South China, Guangdong Provincial Clinical Research Center for Cancer, Sun Yat-sen University Cancer Center, Guangzhou, China; 4 Department of Etiology and Carcinogenesis, National Cancer Center/National Clinical Research Center for Cancer/Cancer Hospital, Chinese Academy of Medical Sciences and Peking Union Medical College, Beijing, China; 5 Department of Mathematical Sciences, University of Copenhagen, Copenhagen, Denmark; 6 Institute of computing technology, Chinese Academy of Sciences, Beijing, China; 7 Cardiac Arrhythmia Center, Department of Cardiology, Fuwai Hospital, National Center for Cardiovascular Diseases, Chinese Academy of Medical Sciences and Peking Union Medical College, Beijing, China; 8 Department of Cardiology, Shanghai Tenth People's Hospital, Tongji University, School of Medicine, Shanghai, China

**Keywords:** Exercise, Accelerometer, Public health

## Abstract

**Objective:**

To explore the association of wearable device-measured moderate-to-vigorous intensity physical activity (MVPA) with cardiovascular disease (CVD) risk in long-term cancer survivors.

**Methods:**

This retrospective analysis involved a prospective cohort of 6109 cancer survivors without CVD from the UK Biobank accelerometry subsample. The MVPA volume is categorised into four groups based on guideline recommendations (0–75 min/week, 75–150 min/week, 150–300 min/week, ≥300 min/week). Cox proportional hazard models are used to investigate the association of MVPA with incident CVD.

**Results:**

Over a median follow-up of 7.88 years, there were 539 incident CVD events (361 incident coronary artery disease (CAD) events, 155 incident heart failure (HF) events, and 109 incident stroke events). Adjusted CVD incidence rates (95% CIs) across MVPA groups (0–75 min/week, 75–150 min/week, 150–300 min/week, ≥300 min/week) were 15.30 (12.90, 18.10), 13.50 (11.00, 16.40), 12.00 (10.20, 14.10) and 9.86 (8.35, 11.60) per 1000 person-years, respectively. Adjusted HRs (95% CI) for CVD, CAD, HF and stroke in the highest MVPA group (≥300 min/week) compared with those in the lowest MVPA group (0–75 min/week) were 0.63 (0.49, 0.80), 0.68 (0.51, 0.91), 0.66 (0.42,1.06) and 0.72 (0.42, 1.23), respectively. For obesity-related cancers, the beneficial effect on CVD was observed when exceeding 300 MVPA min/week (HR 0.54 (0.37–0.81)) compared with the lowest MVPA group.

**Conclusions:**

Findings from the UK Biobank study suggest that longer MVPA durations are associated with reduced CVD risk in cancer survivors, underscoring the potential for physical activity to serve as a key component in cardio-oncology care.

**WHAT IS ALREADY KNOWN ON THIS TOPIC**

- Cardiovascular disease (CVD) is the leading cause of non-cancer mortality among cancer survivors. Despite the known benefits of physical activity (PA) on cardiovascular health in the general population, evidence regarding post-diagnosis PA and CVD risk among cancer survivors is limited.

**WHAT THIS STUDY ADDS**

- From wearable device-measured moderate-to-vigorous intensity PA data in cancer survivors, adhering to PA guidelines (150–300 min per week) and surpassing them (≥300 min per week) were associated with 23% and 37% lower risk of incident CVD, respectively, compared with those with the lowest PA duration (0–75 min per week).

**HOW THIS STUDY MIGHT AFFECT RESEARCH, PRACTICE or POLICY**

- These findings demonstrate that meeting or exceeding 150–300 min per week of MVPA should be encouraged as a means to reduce long-term CVD risk in cancer survivors.

## Introduction

 Cancer survivorship has been increasing with nearly 18 million cancer survivors in the USA in 2022.^1^ Cardiovascular disease (CVD) is the leading cause of non-cancer mortality among long-term cancer survivors,^2 3^ highlighting an urgent need to establish cardioprotective strategies within cardio-oncology care. In 2019, the American Heart Association proposed the Cardio-Oncology Rehabilitation (CORE) model for cardiovascular rehabilitation of cancer survivors, emphasising physical activity (PA) as a cornerstone of the CORE model.^4^ However, there is still limited evidence regarding the effectiveness of post-diagnosis PA on CVD outcomes among cancer survivors.

Prior work has primarily examined the overall survival of prevalent cancers (breast, endometrial, colorectal and prostate cancers),^5^,^8^ rather than specific CVD outcomes. The few available analyses of PA and CVD outcomes involve small sample sizes (less than 500) and focus on a single cancer site,^9^ typically breast cancer and cardiac function.^4 10^ As a result, there is no established guideline for the intensity and duration of PA prescription specifically for cancer survivors to prevent CVD risk. Existing recommendations for cancer survivors are generally based on guidelines for healthy individuals, such as participation in 150 min/week of moderate-intensity or 75 min/week of vigorous-intensity PA.^6 11^ Further evidence regarding the impact of PA on CVD risk in cancer survivors will facilitate PA recommendations within cardio-oncology care.

Numerous cancers are associated with excess body weight, and the International Agency for Research on Cancer (IARC) identified 13 cancer types linked to obesity.^12^ Research suggests that obesity accelerates the progression from normal tissue to invasive malignancy and metastatic disease.^13^ Furthermore, increased body fat may raise the CVD risk in obesity-related cancer patients. Since PA plays a key role in reducing body fat, post-diagnosis PA is thought to improve cancer prognosis by modulating metabolic, inflammatory and immune pathways.^5 14^ In contrast, non-obesity-related cancers may not be as affected by these mechanisms. Given these potential differences, our study further categorised cancer into obesity-related and non-obesity-related groups to better explore how post-diagnosis PA may differentially impact CVD risk in long-term cancer survivors.

Therefore, the present study, using data from a large-scale prospective cohort study, aimed to explore the associations between post-diagnosis PA and CVD risk in long-term cancer survivors.

## Methods

The UK Biobank wrist accelerometry sub-study comprised more than 100 000 adults from 2013 to 2015. The sub-study approved by the UK National Research Ethics Service (No.11/NW/0382) had obtained written informed consent for all participants.^15^ This study was based on the UK Biobank cohort study under application number 91 035. We followed the ‘Strengthening the Reporting of Observational Studies in Epidemiology’ reporting guideline (online supplemental table S8).

### Study population

**Figure 1:**
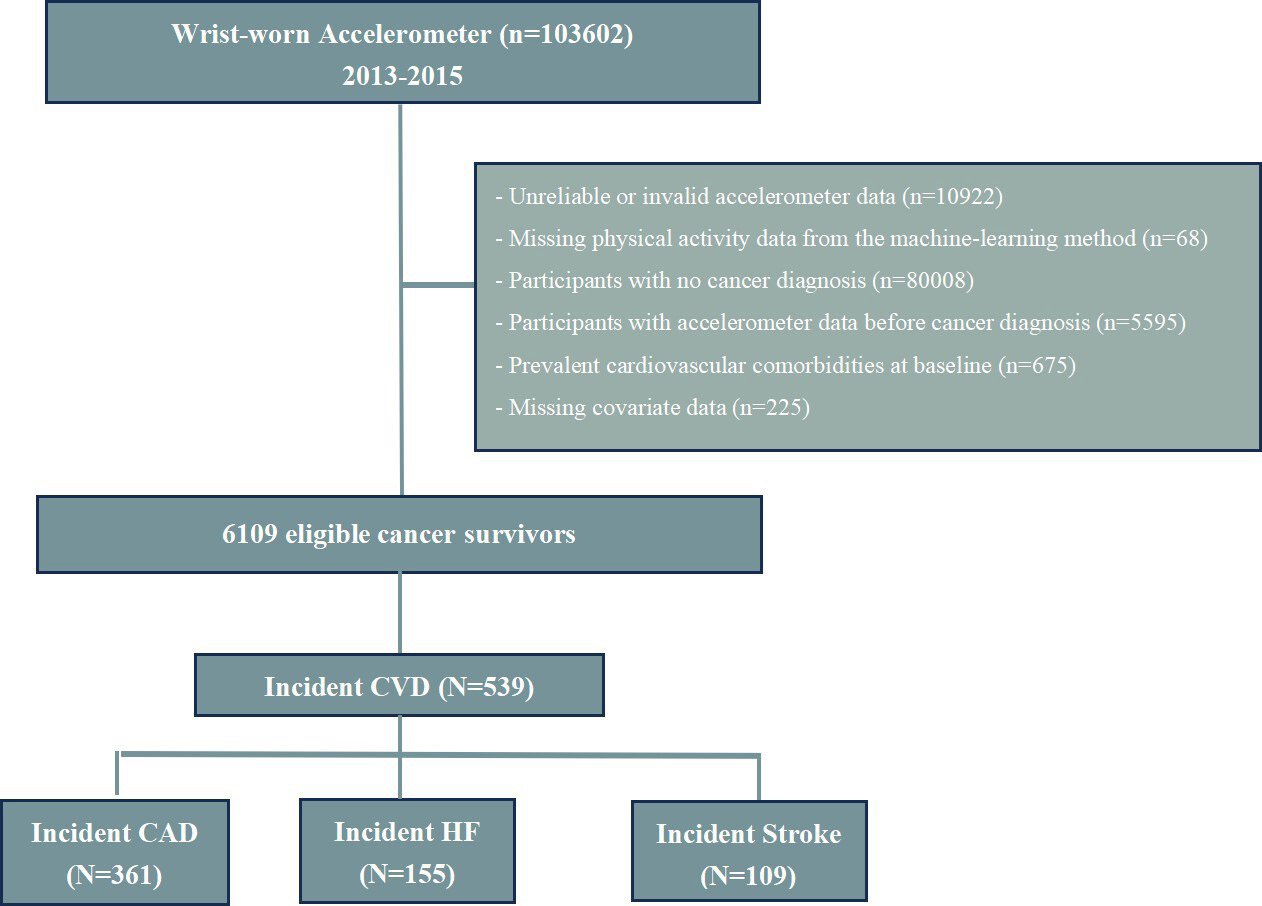
Selection of the study population. The diagram illustrates the number of cancer survivors within the population who engaged in accelerometer-measured physical activity. CVD, cardiovascular diseases; CAD, coronary artery diseases; HF: heart failure.

From the wrist accelerometry substudy activity data, we included only cancer survivors with valid accelerometer data (online supplemental methods). We further excluded participants with baseline CVD (coronary artery disease (CAD) [ICD-10, I21-25], heart failure (HF) [ICD-10, I50] or stroke [ICD-10, I60-I61, I63-I64]) or missing covariates (figure 1, online supplemental figure S1). Cancer data linkage was obtained through national cancer registries. We examined all cancers (C00–C97, except for non-melanoma skin cancer C44, in situ and non-well-defined cancers).^16^ Methods for cancer assessment are provided in the online supplemental methods section. Detailed coding and numbers of site-specific cancers are presented in online supplemental table S1. The sample size justification related to the different endpoints is presented in the online supplemental methods.

Cancer types were further categorised as obesity-related and non-obesity-related based on the IARC criteria^12^ because previous studies on PA have mainly focused on obesity-related cancers. Obesity-related cancers include endometrial, oesophageal, stomach, liver, kidney, multiple myeloma, brain, pancreatic, colorectal, gallbladder, breast, ovarian and thyroid cancers.^17^

### PA assessment

MVPA behaviours were classified using a previously published two-stage machine-learning Random Forest activity classifier developed and validated for use with the UK Biobank^18 19^ (see online supplemental methods). The corresponding MVPA behaviours are detailed in online supplemental table 2). This machine-learning approach was based on the CAPTURE-24 study containing the largest free-living dataset and demonstrated superior performance in practice. Compared with traditional ‘vector magnitude’ methods, the machine-learning approach leverages multiple data features to classify behaviours with greater accuracy.^19^ The MVPA volume was further categorised into four groups based on guideline-recommended weekly MVPA volume (>0 to <75 min/week, 75 to <150 min/week, 150 to <300 min/week and ≥300 min/week).

### Outcome ascertainment

The primary outcome was the occurrence of CVD, including CAD (ICD-10, I21-25), HF (ICD-10, I50) or stroke (ICD-10, I60-I61, I63-I64). The incident CVD information was gathered from self-report, primary care and hospital admission sources. Participants were followed up from the completion of accelerometer wear to the incidence of CVD, loss to follow-up, death or the end of follow-up on 31 December 2022, whichever occurred first.^6^ . Online supplemental tables S3 and S4 contain complete definitions of outcomes and variables, as well as selection rationales.

### Statistical analysis

Complete-case analysis was conducted in this study. Descriptive characteristics were reported as mean (SD) for normally distributed variables or median (IQR) for skewed continuous variables and number and percentage for categorical variables.

Analysis in Model 1 showed an unadjusted HR, while Model 2 was adjusted for age, sex, ethnicity, Townsend index of deprivation, educational levels, smoking status, alcohol intake, diet score, sleep duration, medications (lipid-lowering, antihypertensive, antidiabetic), self-reported parental history of CVD or cancer and time from cancer diagnosis to accelerometer-worn completion. The selection of variables was guided by a directed acyclic graph (DAG), developed through a comprehensive literature review. This process is outlined in detail in online supplemental table S4, with the resulting DAG shown in online supplemental figure S2. Model 2 served as the main analysis.

The dose-response analysis of MVPA duration with outcomes was conducted using restricted cubic splines, with knots positioned at the 10th, 50th and 90th percentiles according to the previous literature,^20^,^22^ and the reference group set to zero minutes per week. For HR analyses, the departure from linearity was assessed by a Wald test examining the null hypothesis that the coefficient of the second spline was equal to zero.^22^ Poisson regression models with log-transformed follow-up time as the offset were used to estimate the crude and adjusted incidence rates across MVPA groups, reported as events per 1000 person-years. Model assumptions were evaluated by calculating the ratio of residual deviance to df for overdispersion and using residual distribution plots to assess the linearity of quantitative predictors. Adjusted incidence rate differences and rate ratios were estimated using g-computation with standard parametric regression models.^23^ Adjusted HR with 95% CI was estimated using Cox proportional hazards regression models. Cox proportionality assumptions were assessed using Schoenfeld residuals, with no observed violations. The cumulative risks of CVD across MVPA groups were compared as adjusted survival curves.^24 25^ We also calculated the 5 year absolute risk across MVPA groups using Fine and Grey competing risks regression models. Exploratory analyses were further conducted in survivors of obesity-related and non-obesity-related cancers, respectively.^12 17^

In sensitivity analysis, we further excluded people with diseases of the circulatory system (I0, I11, I13, I20-I51, I60-I69) at baseline. A landmark analysis was performed by delaying the start of follow-up by 2 years following accelerometer-worn completion to account for potential reverse causality. We employed the Fine and Grey and cause-specific hazard model to account for competing risks (non-CVD deaths as competing risks). Body mass index (BMI) may mediate the relationship between PA and CVD, so it was excluded from the main analysis. However, as BMI could also act as a confounder, an additional analysis was conducted with BMI adjustment. Furthermore, we classified MVPA based on WHO standards: below WHO guidelines (0–150 min), meeting WHO guidelines but below the extended recommendation (150–300 min) and exceeding the extended WHO recommendation (≥300 min) to further validate the robustness of our results. An additional analysis was performed using covariates from the interview closest to the accelerometer assessment. Missing covariate values were imputed using multiple imputation by chained equations (see online supplemental methods and online supplemental tables S5 andS6). Finally, multiplicative and additive interaction (using the ‘interactionR’ package in R) analyses and subgroup analyses were performed. All analyses were performed using R version 4.3.1.

### Equity, diversity and inclusion

The author group is gender balanced, consisting of junior, mid-career and senior researchers from several disciplines, including cardiology, oncology, computing technology and sport. Our study cohort included male and female cancer survivors of different race and ethnicity, but the majority were white.

## Results

### Sample characteristics

The study sample comprised 6109 cancer survivors, with a mean (SD) age of 65.4 (6.9) years (table 1). Of these, 3722 (60.9%) were women, 2387 (39.1%) were men, and 5991 (98.1%) were White individuals. The most common specific cancer types were breast (n=2063, 33.8%), prostate (n=1151, 18.9%), small intestine (n=593, 9.7%), melanoma skin (n=506, 8.3%) and lymphatic and haematopoietic tissue (n=457, 7.5%). Details of the number of cancer survivors by specific types are presented in online supplemental table S1. Cancers were further divided into obesity-related (n=3401, 55.7%) and non-obesity-related (n=2708, 44.3%) categories.

**Table 1:** Sample size and characteristics for UK Biobank Cancer Survivors by guideline-recommended moderate-to-vigorous physical activity

	Total	Moderate-to-vigorous physical activity (min/week)			
		0≤MVPA<75	75≤MVPA<150	150≤MVPA<300	MVPA≥300
n (%)	6109	1315 (21.5)	1046 (17.1)	1768 (29.0)	1980 (32.4)
Age, years, median (IQR)	66.85(61.09, 70.54)	67.60(62.24, 71.28)	67.18(61.70, 71.06)	66.75(61.10, 70.42)	66.10(60.17, 69.87)
Sex, n (%)					
Male	2387 (39.1)	360 (27.4)	299 (28.6)	724 (41.0)	1004 (50.7)
Female	3722 (60.9)	955 (72.6)	747 (71.4)	1044 (59.0)	976 (49.3)
White ethnicity, n (%)	5991 (98.1)	1285 (97.7)	1022 (97.7)	1735 (98.1)	1949 (98.4)
Townsend deprivation index, median (IQR)	−2.57(-3.87,–0.43)	−2.56(-3.86,–0.43)	−2.42(-3.78,–0.29)	−2.67(-3.90,–0.47)	−2.57(-3.87,–0.48)
University education, n (%)	2650 (43.4)	439 (33.4)	394 (37.7)	799 (45.2)	1018 (51.4)
Smoking status, n (%)					
Never	3351 (54.9)	648 (49.3)	579 (55.4)	1003 (56.7)	1121 (56.6)
Previous	2392 (39.2)	551 (41.9)	397 (38.0)	675 (38.2)	769 (38.8)
Current	366 (6.0)	116 (8.8)	70 (6.7)	90 (5.1)	90 (4.5)
Alcohol consumption, n (%)					
Never	1211 (19.8)	362 (27.5)	250 (23.9)	314 (17.8)	285 (14.4)
Moderate	3297 (54.0)	651 (49.5)	553 (52.9)	982 (55.5)	1111 (56.1)
Excessive	1601 (26.2)	302 (23.0)	243 (23.2)	472 (26.7)	584 (29.5)
Diet score, n (%)					
0	663 (10.9)	170 (12.9)	107 (10.2)	192 (10.9)	194 (9.8)
1	2258 (37.0)	514 (39.1)	356 (34.0)	659 (37.3)	729 (36.8)
2	2207 (36.1)	446 (33.9)	413 (39.5)	622 (35.2)	726 (36.7)
3	981 (16.1)	185 (14.1)	170 (16.3)	295 (16.7)	331 (16.7)
Parental history of CVD, n (%)	3458 (56.6)	759 (57.7)	605 (57.8)	998 (56.4)	1096 (55.4)
Parental history of cancer, n (%)	2103 (34.4)	454 (34.5)	367 (35.1)	599 (33.9)	683 (34.5)
Medication for cholesterol, n (%)	870 (14.2)	248 (18.9)	166 (15.9)	243 (13.7)	213 (10.8)
Medication for blood pressure, n (%)	1182 (19.3)	338 (25.7)	233 (22.3)	315 (17.8)	296 (14.9)
Medication for diabetes, n (%)	34 (0.6)	13 (1.0)	7 (0.7)	9 (0.5)	5 (0.3)
Physical measures and biomarkers					
Systolic blood pressure, mmHg, median (IQR)	138.00(125.50, 151.00)	140.00(128.00, 152.00)	139.00(125.00, 151.00)	138.00(126.00, 151.12)	137.00(125.00, 150.50)
Diastolic blood pressure, mmHg, median (IQR)	81.00(74.50, 88.00)	82.50(75.50, 88.50)	80.50(74.00, 88.00)	81.50(74.00, 87.50)	80.50(74.50, 87.50)
Body mass index, kg/m^2^, median (IQR)	26.02(23.56, 29.07)	27.74(24.72, 31.63)	26.47(23.92, 29.65)	25.87(23.40, 28.58)	25.19(22.98, 27.66)
C reactive protein, mg/L, median (IQR)	1.28(0.66, 2.62)	1.84(0.95, 3.70)	1.52(0.77, 3.00)	1.17(0.61, 2.32)	1.02(0.53, 2.05)
eGFR, mL/min/1.73 m2, median (IQR)	95.61([84.65, 101.18)	94.88(82.35, 100.61)	95.52(84.63, 100.70)	95.89(85.40, 101.39)	96.00(85.63, 101.61)
LDL direct, mmol/L, median (IQR)	3.58(3.01, 4.18)	3.59(3.00, 4.26)	3.62(3.06, 4.24)	3.57(3.00, 4.13)	3.56(3.01, 4.13)
Lipoprotein A, nmol/L, median (IQR)	20.05(9.45, 57.91)	23.24(10.52, 63.32)	17.35(8.75, 53.72)	20.20(9.93, 53.84)	19.97(9.19, 58.15)
HDL, mmol/L, median (IQR)	1.45(1.22, 1.75)	1.43(1.19, 1.71)	1.46(1.22, 1.75)	1.45(1.21, 1.75)	1.48(1.25, 1.80)
Triglycerides, mmol/L, median (IQR)	1.47(1.05, 2.10)	1.63(1.14, 2.31)	1.53(1.10, 2.14)	1.47(1.06, 2.06)	1.34(0.97, 1.93)
Glycated haemoglobin (HbA1c), mmol/mol, median (IQR)	35.20(33.00, 37.60)	35.90(33.40, 39.00)	35.30(33.10, 38.00)	35.10(32.80, 37.40)	35.00(32.80, 37.10)
Interval between cancer diagnosis and wrist-worn completion, years, median (IQR)	6.92(3.09, 11.95)	7.13(3.02, 12.20)	7.14(3.08, 12.27)	6.68(3.11, 11.85)	6.84(3.13, 11.67)
Cancers, n (%)					
Non-obesity-related cancers, n (%)	2708 (44.3)	469 (35.7)	379 (36.2)	819 (46.3)	1041 (52.6)
Obesity-related cancers ^*^, n (%)	3401 (55.7)	846 (64.3)	667 (63.8)	949 (53.7)	939 (47.4)
Accelerometer data					
Wear duration, days, median (IQR)	6.91(6.74, 7.00)	6.87(6.59, 7.00)	6.90(6.72, 7.00)	6.91(6.72, 7.00)	6.94(6.82, 7.00)
Sleep, h/d, median (IQR)	8.45(7.64, 9.28)	8.50(7.58, 9.51)	8.49(7.68, 9.34)	8.42(7.57, 9.22)	8.43(7.70, 9.16)
Moderate-to-vigorous physical activity, min/week, median (IQR)	201.00([90.00, 358.80)	32.40(13.20, 53.40)	109.80(93.60, 130.20)	216.00(179.40, 256.35)	450.90(366.00, 583.20)

* Obesity-related cancers include oesophageal, stomach, liver, gallbladder, kidney, multiple myeloma, brain, pancreatic, colorectal, breast, ovarian, endometrial and thyroid cancers.

MVPA, moderate-to-vigorous intensity physical activity.

The median time between cancer diagnosis and completion of accelerometer wear was 6.9 (IQR, 3.1–12.0) years (table 1). The median duration of MVPA was 201.0 (IQR, 90.0–358.8) min per week, with 1768 (29.0%) cancer survivors meeting the standard guideline recommendation of 150–300 min/week, and 1980 (32.4%) meeting the extended recommendations of ≥300 min/week. Individuals with longer MVPA duration were more likely to be younger, male, nonsmokers, have a higher educational level, lower BMI and blood pressure levels, report better diet quality and use fewer medications.

### Incident CVD

**Figure 2:**
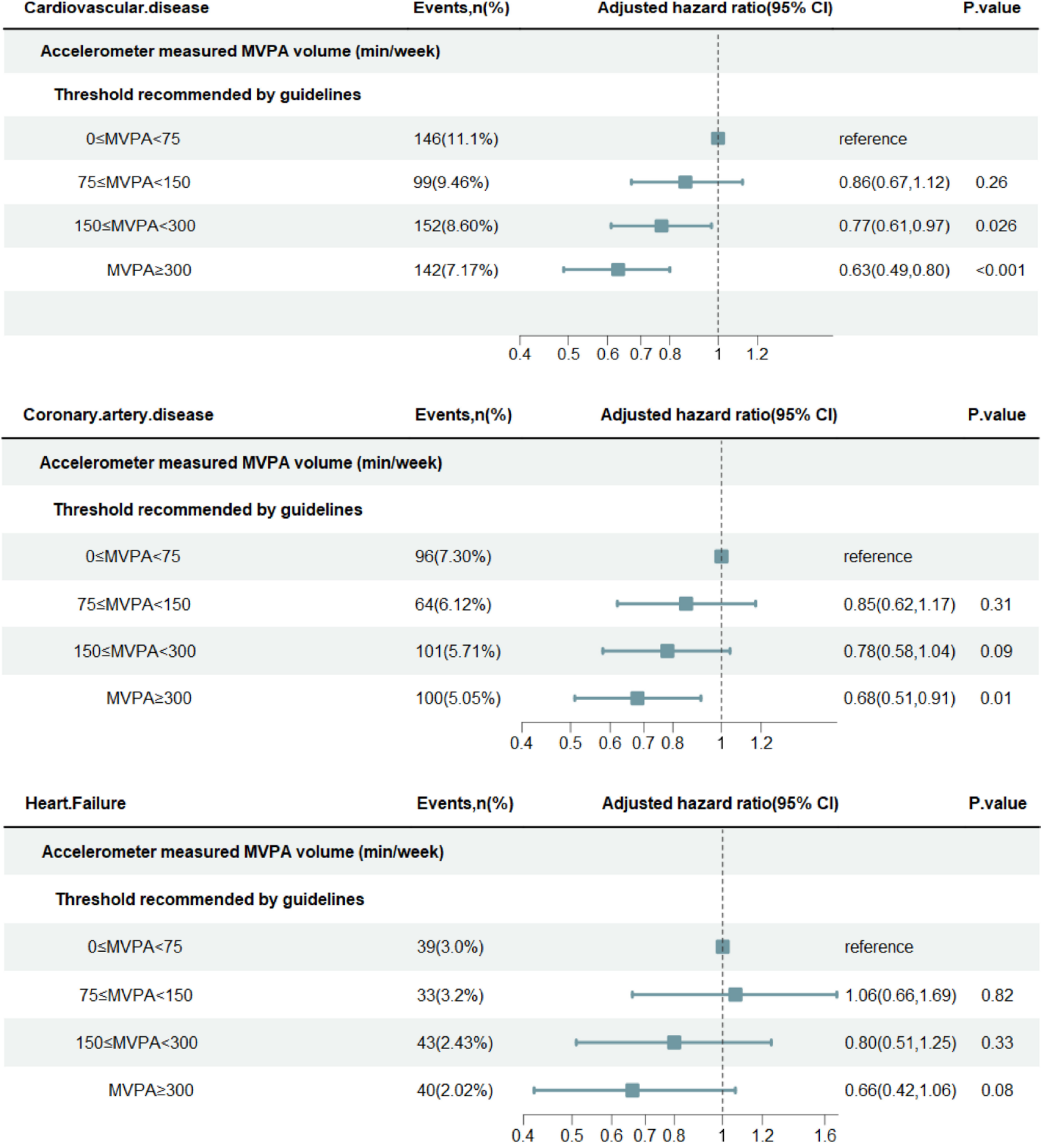
Adjusted HR for incident cardiovascular events by guideline-recommended moderate to vigorous physical activity. The relative risk of incident cardiovascular events. The adjusted HR was estimated using the Cox proportional hazards model, with follow-up years as the timescale. Adjustments were made for age, sex, education, Townsend Deprivation Index, ethnicity (white/non-white), medication (cholesterol, blood pressure or diabetes), smoking status, alcohol consumption, diet score, sleep duration, parental history of CVD or cancer and years since first cancer diagnosis. The solid line represents the adjusted HR, with the ribbon indicating the 95% CI. The grey area denotes the population proportion (units: 30 min/week). MVPA: moderate-to-vigorous intensity physical activity.

During a median follow-up of 8.01 (IQR 7.95, 8.04) years, there were 539 incident CVD events, including 361 incident CAD events, 155 incident HF events and 109 incident stroke events. And 451 participants died from other causes. An inverse near-linear dose-response relationship between MVPA volume and the incidence of CVD was observed (P for nonlinear=0.49), with no maximal threshold for the benefits (online supplemental figure S3). The adjusted rates of CVD incidence (95% CIs) across MVPA groups (0–75 min/week, 75–150 min/week, 150–300 min/week, ≥300 min/week) were 15.30 (12.90, 18.10), 13.5 (11.00, 16.40), 12.00 (10.20, 14.10) and 9.86 (8.35, 11.60) per 1000 person-years, respectively (table 2). Adhering to MVPA within the recommended duration (150–300 min per week) and surpassing it (≥300 min per week) were associated with a 23% (HR, 0.77, 95% CI, 0.61 to 0.97) and 37% (HR, 0.63, 95% CI, 0.49, 0.80) lower hazard of CVDs, respectively, compared with those with the lowest PA duration (0–75 min per week) (figure 2). For specific types of CVD, patients with longer MVPA duration were associated with a reduced risk of CAD (≥300 MVPA min/week vs 0–75 MVPA min/week: HR, 0.68 [0.51, 0.91], p=0.01, S-value=6.64). There was weaker evidence for associations between MVPA and HF (≥300 MVPA min/week vs 0–75 MVPA min/week: HR, 0.66 [0.42,1.06], p=0.08, S-value=3.6) or stroke (≥300 MVPA min/week vs 0–75 MVPA min/week: HR, 0.72 [0.42, 1.23], p=0.23, S-value=2.12). However, the data are still compatible with the potential protective effects of MVPA on these outcomes, as suggested by the direction of the hazard ratios. Adjusted survival curves for CVD and subtypes of CVD across MVPA groups were depicted in online supplemental figure S4. Furthermore, we employed the Fine and Gray competing risks regression model to calculate the 3 and 5 year cumulative CVD risks presented in online supplemental figure S5. Likewise, a higher MVPA volume reduces the CVD incidence across different subgroups.

**Table 2:** Incidence event rate, incidence rate difference, and incidence rate ratio of cardiovascular diseases by guideline-recommended moderate-to-vigorous physical activity

		Accelerometer-derived MVPA cohort				
		Total	0≤MVPA<75(min/week)	75≤MVPA<150(min/week)	150≤MVPA<300(min/week)	300≤MVPA (min/week)
CVD	Event, n	539	146	99	152	142
	Incidence event rate ^*^	11.80 (10.83, 12.85)	15.30 (12.90, 18.02)	12.74 (10.36, 15.51)	11.46 (9.71, 13.44)	9.43 (7.94, 11.12)
	Adjusted incidence event rate ^*,†^		15.30 (12.90, 18.10)	13.50 (11.00, 16.40)	12.00 (10.20, 14.10)	9.86 (8.35, 11.60)
	Adjusted incidence rate difference ^*,†^		Ref.	−1.84 (-5.83, 1.74)	**−3.28 (−6.27，–0.50**)	**−5.43 (-8.61, –2.05**)
	Adjusted incidence rate ratio ^†^		Ref.	0.88 (0.67, 1.14)	**0.79 (0.63, 0.97**)	**0.65 (0.50, 0.85**)
CAD	Event, n	361	96	64	101	100
	Incidence event rate ^*^	7.82 (7.03, 8.67)	9.85 (7.96, 12.05)	8.16 (6.28, 10.42)	7.56 (6.15, 10.42)	6.59 (5.36, 8.02)
	Adjusted incidence event rate^*^,^†^		9.80 (7.98, 12.10)	8.54 (6.65, 11.00)	7.87 (6.46, 9.59)	6.87 (5.63, 8.38)
	Adjusted incidence rate difference*^,†^		Ref.	−1.27 (-4.13, 1.63)	−1.93 (-4.76, 0.79)	**−2.94 (-5.64, –0.26**)
	Adjusted incidence rate ratio†		Ref.	0.87 (0.64, 1.18)	0.80 (0.59, 1.10)	**0.70 (0.51, 0.97**)
HF	Event, n	155	39	33	43	40
	Incidence event rate ^*^	3.31 (2.81, 3.87)	4.00 (2.84, 5.47)	4.12 (2.84, 5.79)	3.15 (2.28, 4.24)	2.58 (1.85, 3.52)
	Adjusted incidence event rate ^*,†^		4.01 (2.89, 5.57)	4.24 (3.01, 5.97)	3.26 (2.42, 4.38)	2.70 (1.96, 3.71)
	Adjusted incidence rate difference ^*,†^		Ref.	0.23 (-1.59, 1.97)	−0.75 (-2.54, 0.79)	−1.31 (-2.77, 0.12)
	Adjusted incidence rate ratio ^†^		Ref.	1.06 (0.66, 1.61)	0.81 (0.52, 1.23)	0.67 (0.44, 1.04)
Stroke	Event, n	109	30	20	29	30
	Incidenceevent rate ^*^	2.32 (1.91, 2.80)	3.07 (2.07, 4.39)	2.49 (1.52, 3.84)	2.12 (1.42, 3.05)	1.94 (1.31, 2.77)
	Adjusted incidence Event Rate ^*, †^		2.92 (2.03, 4.21)	2.62 (1.69, 4.06)	2.22 (1.53, 3.21)	2.06 (1.44, 2.95)
	Adjusted incidence rate difference ^*, †^		Ref.	−0.31 (-1.74, 1.23)	−0.71 (-2.14, 0.57)	−0.86 (-2.19, 0.30)
	Adjusted incidence rate ratio ^†^		Ref.	0.90 (0.50, 1.52)	0.76 (0.41, 1.30)	0.71 (0.39, 1.16)

* per 1000 person-years (95 % CI)

† Adjusted for age, sex, education, Townsend Deprivation Index, ethnicity(white/non-white), medication (cholesterol, blood pressure or diabetes), smoking status, alcohol consumption, diet score, time spent in sleep, parental history of CVD or cancer and years since first cancer diagnosis.

MVPA, moderate-to-vigorous intensity physical activity.

### Subgroup analysis and sensitivity analyses

The CVD benefits remained consistent regardless of cancer type. There was an inverse near-linear relationship between MVPA duration and CVD risk (non-obesity-related cancers: P for nonlinearity=0.51, P for trend=0.035; obesity-related cancers: P for nonlinearity=0.82, P for trend<0.001) (online supplemental figure S6, table 3). For non-obesity-related cancers, the HR (95% CI) for 75–150 min of MVPA per week was 0.65 (0.44–0.97), for 150–300 min of MVPA per week was 0.70 (0.51–0.97) and for exceeding 300 min MVPA/week was 0.64 (0.46–0.88), compared with the lowest MVPA group (0–75 min/week). For obesity-related cancers, the reduced HRs (95% CI) are 0.82 (0.59–1.16) and 0.54 [0.37–0.81] for 150–300 min of MVPA per week and exceeding 300 MVPA minutes per week, respectively.

**Table 3:** HR (95% CI) for incident cardiovascular diseases among the obesity and non-obesity-related cancer participants

		Non-obesity-related cancers					Obesity-related cancers			
	Events/n	UnadjustedHR (95% CI)	P value	Model 2*HR (95% CI)	P value	Events/n	UnadjustedHR (95% CI)	P value	Model 2 aHR (95% CI)	P value
Cardiovascular diseases										
MVPA (min/week)										
0≤MVPA<75	70/469	Ref		Ref		76/846	Ref		Ref	
75≤MVPA<150	38/379	**0.64 (0.43 to 0.95**)	**0.027**	**0.65 (0.44 to 0.97**)	**0.036**	61/667	0.99 (0.70 to 1.38)	0.93	1.06 (0.75 to 1.49)	0.74
150≤MVPA<300	87/819	**0.68 (0.49 to 0.93**)	**0.015**	**0.70 (0.51 to 0.97**)	**0.033**	65/949	0.73 (0.52 to 1.01)	0.058	0.82 (0.59 to 1.16)	0.26
MVPA≥300	101/1041	**0.61 (0.45 to 0.83**)	**0.001**	**0.64 (0.46 to 0.88**)	**0.006**	41/939	**0.46 (0.31 to 0.67**)	**<0.001**	**0.54 (0.37 to 0.81**)	**0.003**
P for trend				0.035					0.001	
Coronary artery diseases										
MVPA (min/week)										
0≤MVPA<75	45/469	Ref		Ref		51/846	Ref		Ref	
75≤MVPA<150	30/379	0.80 (0.50 to 1.26)	0.33	0.81 (0.51 to 1.29)	0.37	34/667	0.82 (0.53 to 1.27)	0.38	0.88 (0.56 to 1.36)	0.55
150≤MVPA<300	55/819	**0.67 (0.45 to 1.00**)	0.049	0.70 (0.47 to 1.05)	0.086	46/949	0.77 (0.52 to 1.15)	0.20	0.87 (0.57 to 1.30)	0.49
MVPA≥300	69/1041	**0.65 (0.45 to 0.95**)	0.027	0.69 (0.47 to 1.03)	0.068	31/939	**0.52 (0.33 to 0.81**)	**0.004**	**0.62 (0.39 to 0.99**)	**0.044**
P for trend				0.125					0.046	
Heart Failure										
MVPA (min/week)										
0≤MVPA<75	21/469	Ref		Ref		51/846	Ref		Ref	
75≤MVPA<150	13/379	0.73 (0.36 to 1.45)	0.36	0.71 (0.35 to 1.43)	0.34	34/667	1.37 (0.73 to 2.60)	0.33	1.45 (0.76 to 2.77)	0.26
150≤MVPA<300	24/819	0.62 (0.34 to 1.11)	0.11	0.64 (0.35 to 1.16)	0.14	46/949	0.91 (0.48 to 1.73)	0.77	1.01 (0.52 to 1.95)	0.98
MVPA≥300	32/1041	0.63 (0.37 to 1.10)	0.11	0.68 (0.38 to 1.22)	0.19	31/939	**0.38 (0.17 to 0.88**)	0.024	0.46 (0.19 to 1.09)	0.077
P for trend				0.362					0.003	
Stroke										
MVPA (min/week)										
0≤MVPA<75	10/469	Ref		Ref		20/846	Ref		Ref	
75≤MVPA<150	6/379	0.72 (0.26 to 1.97)	0.52	0.77 (0.28 to 2.14)	0.62	14/667	0.86 (0.43 to 1.70)	0.66	0.97 (0.48 to 1.94)	0.93
150≤MVPA<300	17/819	0.94 (0.43 to 2.06)	0.88	1.09 (0.48 to 2.43)	0.84	12/949	0.51 (0.25 to 1.05)	0.067	0.58 (0.29 to 1.25)	0.17
MVPA≥300	22/1041	0.94 (0.45 to 1.99)	0.87	1.08 (0.50 to 2.37)	0.84	8/939	**0.34 (0.15 to 0.78**)	0.01	0.43 (0.18 to 1.02)	0.057
P for trend				0.68					0.035	

* Adjusted for age, sex, education, Townsend Deprivation Index, ethnicity(white/non-white), medication (cholesterol, blood pressure or diabetes), smoking status, alcohol consumption (never/normal/excessive), diet score (0,1,2, or 3), time spent in sleep, parental history of CVD or cancer and years since first cancer diagnosis.

MVPA, moderate-to-vigorous intensity physical activity.

The inverse association between MVPA duration and CVD risk remained consistent across various subgroups, including age of cancer diagnosis (<60, ≥60), sex (male, female), educational background (university degree or not), smoking status (never, current or previous), alcohol intake (<median, ≥median), sleep duration (<7 hours/day, ≥7 hours/day), diet score (0–1, 2–3), hypertension (no or yes), diabetes (no or yes), BMI (<25 kg/m2, ≥25 kg/m2), low-density lipoprotein-cholesterol (<3.4 mmol/L, ≥3.4 mmol/L) and triglycerides (<1.7 mmol/L, ≥1.7 mmol/L) (online supplemental table S10). We did not observe statistically significant additive or multiplicative interactions for any other risk factors (online supplemental table S10).

In sensitivity analyses, adjusting for BMI (online supplemental figure S7), excluding people with diseases of the circulatory system (online supplemental figure S8), excluding the first 2 years of follow-up (online supplemental figure S9), accounting for competing risks from other causes of death (online supplemental table S10), analysing the total case sample, comprising 6334 individuals in the cohort (online supplemental figure S10), using covariates from the interview closest to the accelerometer assessment (online supplemental figure S11) and classifying MVPA based on WHO guidelines (<150 min/week, 150–300 min/week and ≥300 min/week) (online supplemental figure S12) yielded similar results with the main analysis.

## Discussion

In this prospective cohort study of 6109 cancer survivors, we observed an inverse near-linear dose-response relationship between the MVPA volume and the incidence of CVD, with no maximal threshold. Adhering to MVPA within the recommended duration (150–300 min per week) and surpassing it (≥300 min per week) were associated with a 23% and 37% lower risk of CVD, respectively, compared with those with the lowest PA duration (0–75 min per week). The primary reduction in CVD risk was observed in CAD risk. The competing risk models yielded similar results, with slightly weaker effect sizes in the Fine–Gray model. For obesity-related cancers, the beneficial effect on CVD was observed with MVPA exceeding 300 min per week.

Previous research has shown substantial all-cause mortality benefits associated with high levels of PA across various cancers, including bladder, colon, endometrial, haematopoietic, breast, prostate and melanoma cancers.^7^ However, there is still limited evidence regarding the effectiveness of PA on CVD outcomes. Jones *et al* demonstrated a decreased CVD risk with increasing recreational PA levels in non-metastatic breast cancer survivors.^9^ The evidence for other cancers was insufficient. Previous studies mainly relied on questionnaires to assess PA levels,^26^ which are limited in providing a comprehensive evaluation^14^ and may introduce potential biases and misclassification.^27^ In contrast, in the present study, we use wrist accelerometer devices to explore the effect of PA on a broad range of cancer types, offering a more objective measurement in a real-world setting.^18 19 28^

PA is an important modifiable risk factor for obesity. Post-diagnosis PA could improve the overall survival of breast, colorectal and prostate cancer survivors.^6^ However, no comprehensive analysis has been conducted to examine the CVD benefits of post-diagnosis PA in these populations. Our findings suggest that obesity-associated cancer survivors could benefit more from extending the duration beyond the recommendation (≥300 min/week) in terms of cardiovascular events. A growing number of studies have confirmed that this level of PA can be achieved via appropriate supervision.^4 29^ For non-obesity-related cancer survivors, engaging in PA within the guideline recommendation (150–300 min/week) can also help lower the CVD risk. The present analysis suggests that sufficient PA can reduce CVD risk regardless of cancer type.

Several pathologic mechanisms may explain how PA reduces the CVD risk among cancer survivors. Cancer and CVD share common risk factors, metabolic pathways, hormonal alterations and systemic inflammation.^30^ PA could mitigate CVD risk by reducing chronic inflammation and hormonal disruptions and enhancing insulin sensitivity and immune surveillance.^5^ The benefits of PA on obesity-related cancers might also be mediated through weight loss and obesity-related metabolic abnormalities. Additionally, it can improve peak aerobic fitness (Vo2), a critical cardiovascular risk marker in cancer survivors,^31^ counteract other risk factors like sedentary lifestyles,^32^ alleviate the side effects of cancer treatments and decrease certain treatment-related complications.^31^

### Clinical implications

Our research aligns with the third American Cancer Society guideline recommendations, suggesting that cancer survivors should engage in regular PA and aim to meet or exceed 150–300 min per week of MVPA when feasible.^6^ Our study further expands guideline knowledge on the effects of PA on CVD. Given the diversity in cancer diagnoses and treatments, survivors should personalise activity levels to their abilities while avoiding inactivity.

## Limitations

First, given the median 7 year survival from the initial diagnosis to wrist accelerometer, our study may mainly include patients with less-aggressive tumours, who are more prone to CVD. Second, the period-specific HRs have a built-in bias due to the differential depletion of susceptible individuals over time. Third, despite adjustments for key confounders such as smoking status and alcohol intake, the potential for residual confounding cannot be ruled out. Additionally, measurement errors in self-reported smoking and alcohol consumption might lead to misclassification and incomplete adjustment. Furthermore, based on our sample size justification, smaller effect sizes for stroke and HF may have gone undetected, necessitating cautious interpretation of these findings. Large-scale randomised controlled trials are needed to confirm our results.

Lastly, the accelerometer data was collected over a single 7 day period at baseline, which may not fully capture long-term habitual PA patterns. Variability in MVPA levels may influence some confounders. However, previous studies have shown high consistency in repeated MVPA measurements (intraclass correlation coefficients between 0.76 and 0.90) over 2 months to 4 years.^33^,^36^ Additionally, lifestyle behaviours recorded approximately 5.6 years earlier have been shown to remain relatively stable. Therefore, these limitations are unlikely to impact the validity of our conclusions substantially.

## Conclusions

A longer duration of MVPA was associated with a lower incidence of CVD in cancer survivors, primarily reducing CAD risk. These findings provide valuable insights for clinical guidance on the intensity and duration of PA in cardio-oncology care.

## Supplementary material

10.1136/bjsports-2024-108734 online supplemental file 1

## Data Availability

This study was conducted based on the UK Biobank cohort study under application number 91035.
