# Supplementary material for: Wearable device-measured physical activity and incident cardiovascular disease in cancer survivors
Source: Br J Sports Med. 2025 Mar 11;59(10):e108734. doi: 10.1136/bjsports-2024-108734 (PMC12171509; doi:10.1136/bjsports-2024-108734)
Supplement: online supplemental file 1 [file bjsports-59-10-s001.docx]

**Supplementary Document**

**Supplementary Methods**

**Accelerometer-measured physical activity**

The UK Biobank, a large-scale prospective study, enrolled half a million participants aged 37–73 from 2006 to 2010. All participants provided written informed consent and study approval was obtained from the National Health Service and the National Research Ethics Service. Between February 2013 and December 2015, randomly selected individuals with valid email addresses were invited for a week-long accelerometer monitoring study. Of the 236,519 participants contacted, 106,053 (45% acceptance rate) agreed to wear the activity monitor^1^. Instructions were to wear the accelerometer on the dominant wrist immediately after receiving it and to continue normal activities. It was to be worn non-stop for seven days, after which it was to be returned to the coordinating center in a pre-paid envelope.

Upon receiving the returned accelerometers, the researchers processed the raw triaxial acceleration data. This involved extracting and calibrating the data, identifying and addressing highlight interrupts and invalid values, and resampling. They then filtered out gravity and sensor noise. Data was aggregated into five-second epochs to assess physical activity intensity distribution. Periods of non-wear, identified as stationary episodes of 60 minutes or more, were excluded. To handle non-wear segments, the team imputed data using averages of similar time-of-day vector magnitudes and intensity distributions from different measurement days.

**Machine-learning algorithm**

Moderate-to-vigorous physical activity (MVPA) behaviors were classified using a machine learning-based method developed by Walmsley et al^2^. This model was built using data from the CAPTURE-24 study, which involved 152 adults in Oxford, UK, between 2014 and 2015. Participants continuously wore wrist-worn accelerometers, carried a camera during waking hours, and kept time-use diaries. Researchers annotated the accelerometer data with detailed activity labels from the Compendium of Physical Activities, using images and diary entries for accuracy^3^. These fine-grained labels were then mapped into broader categories: sleep, sedentary behavior, light physical activity, and MVPA.

Using the labeled data from CAPTURE-24, a machine-learning model combining Random Forests and Hidden Markov Models (HMM) was trained to classify accelerometer data from 103,712 UK Biobank participants. The model categorized behaviors into 30-second intervals as sleep, sedentary behavior, light physical activity, or MVPA.

The Random Forest model classified each time window based on accelerometer features such as intensity, frequency, and duration. The Random Forest model was built with 100 decision trees, where the trees were trained with randomness (bootstrap aggregation or bagging) to reduce variance. Additionally, a balanced Random Forest was used to address class imbalance (e.g., sleep being far more common than MVPA). This was done by ensuring that rare behaviors were sampled equally during the training process. Since human behavior typically follows sequential patterns, the Hidden Markov Model (HMM) was applied to refine these classifications by accounting for temporal dependencies. This corrected unlikely transitions, such as abruptly jumping from sleep to MVPA without intermediate activities. The HMM smoothed out improbable sequences by using transition probabilities derived from the data, ensuring that the final classifications aligned with typical human behavior patterns.

Model performance was evaluated using leave-one-participant-out cross-validation, yielding a mean per-participant accuracy of 88% (95% CI 87% to 89%) and a mean Cohen’s kappa of 0.80 (95% CI 0.79 to 0.82) across all behavior types^2,4^.

**Sample size justification**

In designing an observational cohort, we defined participants engaging in at least 150 minutes of moderate-to-vigorous physical activity (MVPA) per week as the higher-activity group, in accordance with established guidelines. Those below this threshold were categorized as the "lower-activity" group. According to a previous study, 63% of cancer survivors in the UK Biobank wrist accelerometry substudy meet the recommended MVPA duration^5^. Based on prior literature, the incidence rates for cardiovascular outcomes among cancer survivors in UK Biobank are as follows: Cardiovascular events overall: 21.5%; Coronary artery disease: 7.8%; Stroke: 2.7%; Heart failure: 4.3%^6^. Assuming that physical activity could reduce the incidence in the higher-activity group by 30% compared to the lower-activity group, we used PASS 2024 software to calculate the required sample sizes for a power of 90% (1-β = 0.90) with α = 0.05. The estimated sample sizes were: for cardiovascular events: N1: N2 = 1.7, N1 = 1054, N2 = 620, for coronary artery disease: N1: N2 = 1.7, N1 = 3360, N2 = 1976, for stroke: N1: N2 = 1.7, N1 = 10127, N2 = 5957, for heart failure: N1: N2 = 1.7, N1 = 6340, N2 = 3729. For a power of 80% (1-β = 0.90) with α = 0.05, the estimated sample sizes were: for stroke: N1: N2 = 1.7, N1 = 7565, N2 = 4450, for heart failure: N1: N2 = 1.7, N1 = 4745, N2 = 2791.

For this study, we included all eligible participants from the UK Biobank database, totaling 6109 individuals. This sample size provides sufficient power to assess cardiovascular events and coronary artery disease. However, for stroke and heart failure, our sample size is insufficient to detect a statistically significant association if the effect size is smaller than 30%.

**Multiple imputation for missing data**

Missing covariate data were addressed using multiple imputation by chained equations in R software (version 4.2.2), assuming that the missing data were at random^7^. The covariates included in the imputation models were listed in Supplementary data online, Table 5, including all variables used in the analysis models plus candidate predictors, and outcome variables. To reduce random error, we imputed 25 datasets and then combined estimates using Rubin's rules. The imputed overall samples showed similar baseline characteristics to the complete samples (Supplementary data online, Table 6).

The covariates included in the imputation models were Age (Continuous; Square root transformed), Sex(dichotomous), Ethnicity(dichotomous), Townsend deprivation index(continuous), University education(dichotomous), Smoking status (ordinal), Alcohol intake (ordinal), Diet score (ordinal), Sleep time (continuous), Physical activity time (continuous), Systolic Blood Pressure (continuous; log transformed), Body mass index (continuous; log transformed), C-reactive protein (continuous; log transformed), eGFR (continuous; log transformed), HDL(continuous; log transformed, LDL (Continuous; Square root transformed), Lipoprotein(a) (Continuous; Square root transformed), Triglycerides (continuous; log transformed), HbA1c(continuous; log transformed), Interval between cancer diagnosis and wrist-worn completion (Continuous; Square root transformed), Medication for lipid, diabetes and hypertension (dichotomous), Death event (dichotomous), Primary CVD event (dichotomous), Primary CAD event (dichotomous), Primary HF event (dichotomous),Primary Stroke event (dichotomous), Primary CVD event date (Continuous; cumulative hazard rate transformed), Primary CAD event date (Continuous; cumulative hazard rate transformed), Primary HF event date (Continuous; cumulative hazard rate transformed), Primary Stroke event date (Continuous; cumulative hazard rate transformed)

**Inclusion criteria for accelerometer-derived MVPA cohort:**

1. **Cancer Diagnosis:**

Cancer data linkage was obtained through national cancer registries. For England and Wales, cancer diagnosis data were provided by the Medical Research Information Service, based at the NHS Information Centre. For Scotland, cancer diagnosis data were provided by the Information Services Division, which is part of the NHS Scotland. Cancer registry data (Category 100092) are considered the gold standard for ascertaining cancer outcomes in the UK. We utilized data on age at diagnosis (data field 40008) and cancer diagnosis coded by the International Classification of Diseases-10 [ICD-10] (data field 40006) to determine the cancer status. The cohort consists of patients diagnosed with cancer (C00-C97, excluded in situ, nonmelanoma skin cancer, and non−well-defined cancers) based on national cancer registries at baseline.

1. If a participant was diagnosed with cancer at the same site several times, only the first

diagnosis is counted.

1. If a participant was diagnosed with cancer at different sites on different dates, only the first diagnosis is counted.
2. If a participant was diagnosed with cancer at different sites on the same date, both diagnoses are counted.
3. **Participants with valid accelerometer data**

The quality control for accelerometer data must meet the following conditions:

1. Accumulate at least 72 hours of data spanning multiple days, with data recorded for each

hour of the 24-hour cycle.

1. Provide dataset sizes from the accelerometer within the expected normal range, indicating

proper function.

(3) Demonstrate uninterrupted recording periods, with no data gaps.

(4) Supply ample raw data for robust calibration processes.

(5) Ensure adequate data for calibration using the participant's own stationary data, with none

of the three sensor axes exceeding a +/- 300 milli-gravity range.

**Exclusion criteria for accelerometer-derived MVPA cohort:**

1. Withdraw from the UK Biobank.

2. Prevalent CVD at baseline

(1) Heart failure

Congestive heart failure; Left ventricular failure; Heart failure, unspecified

(2) Ischaemic heart diseases

Angina pectoris; Acute myocardial infarction; Subsequent myocardial infarction;

Certain current complications following acute myocardial infarction;

Other acute ischaemic heart diseases; Chronic ischaemic heart disease

(3) Stroke

Subarachnoid haemorrhage; Intracerebral haemorrhage

Other nontraumatic intracranial haemorrhage; Cerebral infarction;

Stroke, not specified as haemorrhage or infarction

3. Prevalent cardiovascular comorbidities at baseline*

CVD was defined as disease of the circulatory system, excluding hypertension, and diseases of arteries, and lymph. The ICD-10 codes included were I0, I11, I13, I20-I51, I60-I69.

*The criterion for exclusion applies only in the sensitivity analysis

**Table S1. Coding and number of site-specific cancers in the cohort.**

| **Cancer site** | **ICD-10** | **Number (%)** |
| --- | --- | --- |
| **All cancers**  **(excl. C44 non-melanoma skin cancer)** | C00-C97 |  |
| **Head and Neck** | C00-14, C30-32 | 137 (2.2) |
| **Gastrointestinal** | C15-C26 | 28 (0.5) |
| **esophagus** | C15 | 36 (0.6) |
| **Stomach** | C16 | 18 (0.3) |
| **Small intestine** | C17 | 593 (9.7) |
| **Colorectal** | C18-C20 | 137 (2.2) |
| **Anal** | C21 | 20 (0.3) |
| **Hepatobiliary tract** | C22-C24 | 10 (0.2) |
| liver | C22 | 5 (0.1) |
| Gallbladder | C23 | 1 (0.0) |
| **Pancreas** | C25 | 12 (0.2) |
| **Lung (including trachea and bronchus)** | C33-C34 | 55 (0.9) |
| **Melanoma skin** | C43 | 506 (8.3) |
| **Breast** | C50 | 2063 (33.8) |
| **Cervical** | C53 | 78 (1.3) |
| **Uterus** | C54-55 | 250 (4.1) |
| Endometrium | C54 | 245 (4.0) |
| **Ovary** | C56 | 139 (2.3) |
| **Prostate** | C61 | 1151 (18.9) |
| **Testicular** | C62 | 84 (1.4) |
| **Kidney** | C64-C65 | 129 (2.1) |
| **Bladder** | C67 | 107 (1.8) |
| **Brain and central nervous system** | C70-C72 |  |
| Brain | C71 | 9 (0.1) |
| **Thyroid** | C73 | 91 (1.5) |
| **Lymphatic and hematopoietic tissue** | C81–C96 | 457 (7.5) |
| Non-Hodgkin lymphoma | C82-85 | 250 (4.1) |
| Multiple myeloma | C90 | 56 (0.9) |
| Leukaemia | C91-95 | 112 (1.8) |
| **Other** | C37-C39, C40-C42, C45-C49, C51-C52, C57-C58, C60, C63, C66, C68-C69, C73-C76, C97 | 145 (2.4) |

**Table S2. Assignment of fine-grained camera image annotations from the Compendium of Physical Activities to broad Moderate to vigorous physical activity behaviors.**

**Moderate to vigorous physical activity behaviors：**

transportation; private transportation;1010 bicycling

transportation; walking; 17165 walking the dog

occupation; interruption;17133 walking upstairs

leisure; miscellaneous; walking;17031 loading /unloading a car implied walking

home activity; child/elderly/pet care; child care; 5181 walking and carrying child

home activity; lawn and garden; gardening service; 8050 digging spading filling garden compositing

leisure; sports; miscellaneous; 17082 hiking or walking at a normal pace through fields and hillsides

home activity; lawn and garden; tree and shrub service; 8025 clearing light brush thinning gardens

home activity; lawn and garden; lawn care service;8165 raking lawn

occupation; interruption; walking;11795 walking on the job and carrying light objects such as boxes or pushing trolleys

leisure; sports; water activities;18070 canoeing/rowing

home activity; lawn and garden; lawn care service;8080 laying crushed rock

leisure; sports; gymnasium and athletics; athletics;12150 running

leisure; sports; ball games;15235 football or baseball playing catch

home activity; miscellaneous; standing;5146 standing packing/unpacking household items occasional lifting

home activity; lawn and garden; gardening service;8245 gardening/picking up fruits vegetables flowers

leisure; miscellaneous; walking;17105 pushing a wheelchair non-occupational

leisure; sports; ball games; 15680 tennis doubles

home activity; lawn and garden; gardening service; 8192 shoveling dirt or mud

leisure; sports; miscellaneous;17082 hiking or walking at a normal pace through fields and hillsides

leisure; dancing;3010 ballet modern or jazz general rehearsal or class

leisure; sports; conditioning;2060 health club exercise

leisure; sports; ball games;15690 tennis singles

home activity; lawn and garden; lawn care service;8095 mowing lawn

occupation; public admin/education/health; health;11615 nursing patient care

occupation; miscellaneous; 11615 (generic) standing lifting items continuously with limited walking

leisure; miscellaneous; 17031 loading /unloading a car implied walking

leisure; sports; conditioning; 2019 bicycling stationary RPM/Spin bike class

home activity; leisure; activities for maintenance of a household; with animals;5192 walking/running playing with animals active periods

home activity; lawn and garden; gardening service;8192 shoveling dirt or mud

home activity; household chores; house cleaning; furniture; 5020 cleaning heavy such as car/windows/garage

leisure; sports; conditioning; 2048 elliptical trainer

occupation; interruption; 11795 walking on job and carrying light objects such as boxes or pushing trolleys

leisure; sports; conditioning; 2050 resistance training

leisure; sports; conditioning; 2070 rowing stationary ergometer

leisure; recreation; outdoor; 5175 walking/running playing with child(ren)

leisure; sports; conditioning; 2010 bicycling stationary

leisure; sports; conditioning; 2120 water aerobics water calisthenics water exercise

home activity; home repair; outdoor; 6020 automobile body work

occupation; agriculture/forestry/fishing; 11192 taking care of animals

leisure; miscellaneous; standing; 21017 standing child care only active periods

leisure; sports; conditioning; 2065 stair-treadmill ergometer general

home activity; household chores; washing/ironing/mending clothes;5092 washing clothes by hand (with or without hanging wash)

leisure; miscellaneous; walking; 17133 walking upstairs

transportation; walking;12150 running

occupation; interruption; walking; 17070 walking downstairs

home activity; household chores; house cleaning; floors; 5140 sweeping garage sidewalk or outside of house

occupation; interruption; walking; 17133 walking upstairs

occupation; agriculture/forestry/fishing; 11540 shoveling digging ditches

occupation; construction; 11050 carrying heavy loads

transportation; walking; 17250 walking as the single means to a destination not to work or class

leisure; miscellaneous; walking; 17070 descending stairs

home activity; miscellaneous; walking; 5121 walking with moving and lifting loads such as bikes and furniture

transportation; walking; 17270 walking as the single means to work or class (not from)

| Table S3. Definitions of covariates and outcomes. | | | |
| --- | --- | --- | --- |
| Disease | **Data field ID** | **ICD-10** | **Notes** |
| Prevalent Disease | | |  |
| Cardiovascular diseases* | 131270-131284;131288;131292;131296-131356;131360-131378 | I0, I11, I13, I20-I51, I60-I69. | CVD was defined as diseases of the circulatory system, excluding hypertension, and diseases of arteries, and lymph. |
| Primary outcome | | |  |
| Cardiovascular disease | 131296; 131298; 131300; 131302; 131304; 131306; 131360; 131362; 131364; 131366; 131368; 131354 | I20-I25; I60-I64; I50 |  |
| Secondary outcome | | |  |
| Coronary artery disease | 131296; 131298; 131300; 131302; 131304; 131306 | I20.0;I20.1;I20.8;I20.9; I21.0; I21.1; I21.2; I21.3; I21.4; I21.9; I22.0; I22.1; I22.8; I22.9; I23; I23.0; I23.1; I23.2; I23.3; I23.5; I23.6; I23.8; I24.0; I24.1; I24.8; I24.9; I25.0; I25.1; I25.2; I25.3; I25.4; I25.5; I25.6; I25.8; I25.9; |  |
| Stroke | 131360; 131362; 131364; 131366; 131368 | I60.0; I60.1; I60.2; I60.3; I60.4; I60.5; I60.6; I60.7; I60.8; I60.9; I61.0; I61.1; I61.2; I61.3; I61.4; I61.5; I61.6; I61.8; I61.9; I62.1; I61.9; I63.0; I63.1; I63.2; I63.3; I63.4; I63.5; I63.6; I63.8; I63.9; I64; |  |
| Heart failure | 131354 | I50.0; I50.1; I50.9; |  |
| Covariates |  |  |  |
| Age | 90011, 34, 52 |  | Participants  entered the study at the end of  accelerometer wear. |
| Sex | 31 |  |  |
| Ethnicity | 21000 |  | Categorized as white/ non-white |
| education | 6138 |  | Categorized according to the access to higher education (college/university degree). |
| Townsend deprivation index | 189 |  | Townsend Deprivation Index of  address at the time of UKB baseline  assessment. |
| Smoking status | 20116 |  |  |
| Alcohol consumption | 4407,4418,4429,4440,4451,4462,  1568,1578,1588,1598,1608,5364 |  | Moderate consumption was considered >0 to <= 20 g/day for women  and >0 and <= 30g/day for men and we coded alcohol consumption as never, moderate, or excessive. |
| Diet score | 1289,1299,1309,1329,1339,1349,1359,1369,1379,1389 |  |  |
| Sleep duration | 90001 |  | Derived using machine-learning  methods described in the main text. |
| Interval between cancer diagnosis and wrist-worn completion | 90001, 40005 |  | The interval between the initial cancer diagnosis and the completion of accelerometer wear. |
| Parental history of CVD or Cancer | 20110, 20107 |  |  |
| Medication for diabetes,  cholesterol or blood  pressure | 6177, 6153 |  |  |
| Note: ICD: international classification disease.  *The criterion for exclusion applies only in the sensitivity analysis. | | |  |

**Table S4. Rationale for selection of covariates**

**Strength of associations seen in observational studies and systematic reviews between covariates and cancer survival and cardiovascular diseases?**

| **Covariates** | | **Cancer survival** | | | | | | | | | | | | | | | | | | **Cardiovascular diseases** | | **References** | |
| --- | --- | --- | --- | --- | --- | --- | --- | --- | --- | --- | --- | --- | --- | --- | --- | --- | --- | --- | --- | --- | --- | --- | --- |
|  |  | **Head and neck** | | **Breast** | | **Prostate** | | **Kidney** | | **Bladder** | | **Digestive system** | | **Hematopoietic** | | **Endometrium** | | **Ovarian** | |  |  |  |  |
| **Social factors** | | | | | | | | | | | | | | | | | | | | | | | |
| age | | All ++ | | | | | | | | | | | | | | | | | | ++ | | 32-35 | |
| sex | | All ++ | | | | | | | | | | | | | | | | | | ++ | | 34-37 | |
| Family history | | All ++ | | | | | | | | | | | | | | | | | | ++ | | 65 | |
| ethnicity | | All ++ | | | | | | | | | | | | | | | | | | ++ | | 34-35;  38-40 | |
| Socia-economic class | | All ++ | | | | | | | | | | | | | | | | | | ++ | | 35; 41-42 | |
| Education | | All ++ | | | | | | | | | | | | | | | | | | ++ | | 35; 43-44 | |
| **Healthy behaviors** | | | | | | | | | | | | | | | | | | | | | | | |
| Smoking status | | ++ | | ++ | | ++ | | NA | | NA | | ++ | | NA | | NA | | ++ | | ++ | | 43-51; 57 | |
| Alcohol consumption | | + | | NA | | NA | | NA | | NA | | + | | + | | NA | | NA | | ++ | | 51-53; 57 | |
| Diet | | + | | ++ | | + | | NA | | NA | | ++ | | NA | | NA | | +/none | | ++ | | 51,53-55; 57 | |
| Sleep duration | | NA | | None | | NA | | NA | | NA | | NA | | NA | | NA | | NA | | ++ | | 56, 57 | |
| **Medications** | | | | | | | | | | | | | | | | | | | | | | | |
| Blood Pressure | | NA | | ++ | | NA | | NA | | NA | | + | | NA | | NA | | + | | ++ | | 57-60 | |
| lipids | | NA | | ++ | | + | | NA | | NA | | ++ | | NA | | ++ | | ++ | | +++ | | 60-61; 57 | |
| Diabetes | | NA | | +++ | | NA | | NA | | NA | | +/none | | NA | | NA | | + | | ++ | | 60, 62-63 | |
| **Obesity-related factors** | | | | | | | | | | | | | | | | | | | | | | | |
| BMI | | + | | +++ | | + | | + | | ++ | | ++ | | none | | + | | + | | ++ | | 5, 10, 60, 64 | |
| obesity | | + | | +++ | | ++ | | + | | + | | +++ | | + | | ++ | | + | | +++ | | 12 | |
| Cancers with ≥100 survivors in the cohort (excluding Melanoma skin cancers due to limited studies).  The strength of the association between the covariates and cancer survival and cardiovascular diseases was categorized in the following way:  “++” showed that ≥3 studies reported a significant positive association. “+” was indicated that 1-2 studies showed a significant positive association.  “+++” was labeled for causal associations.  Data with insufficient evidence were labeled as NA, weak negative correlations as “-”, and no association as none. | | | | | | | | | | | | | | | | | | | | | | | |

**Table S5. Missing Percentage and Reasons for Variables in Cohort**

| **Class** | **Variable** | **Missing Percentage** | **Missing reasons** | **Types** |
| --- | --- | --- | --- | --- |
| **Social factors** | Age | 0.00 |  | Continuous; Square root transformed |
|  | Sex | 0.00 |  | Dichotomous |
|  | Ethnicity | 0.43 | Do not know / Prefer not to answer | Dichotomous |
|  | Townsend deprivation index | 0.03 | — | Continuous |
|  | University education | 0.52 | Prefer not to answer | Dichotomous |
| **Health Behaviors** | Smoking status | 0.27 | Prefer not to answer | Ordinal |
|  | Alcohol intake | 0.06 | Prefer not to answer | Ordinal |
|  | Diet score | 1.41 | Do not know / Prefer not to answer | Ordinal |
|  | Sleep time | 0.0 |  | Continuous |
|  | Physical activity time (MVPA) | 0.0 |  | Continuous |
| **Physical Measures**  **and Biomarkers** | Systolic Blood Pressure | 0.13 | — | Continuous; Log transformed |
|  | Diastolic Blood Pressure | 0.13 | — | Continuous; Log transformed |
|  | Body mass index | 0.17 | — | Continuous; Log transformed |
|  | C-reactive protein | 6.62 | — | Continuous; Log transformed |
|  | eGFR | 6.41 | — | Continuous; Log transformed |
|  | HDL | 14.1 | — | Continuous; Log transformed |
|  | LDL | 6.54 | — | Continuous; Square root transformed |
|  | Lipoprotein(a) | 23.9 | — | Continuous; Square root transformed |
|  | HbA1c | 6.35 | — | Continuous; Log transformed |
|  | Triglycerides | 6.43 | — | Continuous; Log transformed |
| **history& Medications** | Interval between cancer diagnosis and wrist-worn completion | 0.0 |  | Continuous; Square root transformed |
|  | Parental history of CVD or cancer | 0.0 |  | Dichotomous |
|  | Medication for lipid, diabetes, and hypertension | 1.04 | Do not know / Prefer not to answer | Dichotomous |
| **Outcomes** | Death event | 0.00 |  | Dichotomous |
|  | Primary CVD event | 0.00 |  | Dichotomous |
|  | Primary CAD event | 0.00 |  | Dichotomous |
|  | Primary HF event | 0.00 |  | Dichotomous |
|  | Primary Stroke event | 0.00 |  | Dichotomous |
|  | Primary CVD event date | 0.00 |  | Continuous; cumulative hazard rate transformed |
|  | Primary CAD event date | 0.00 |  | Continuous; cumulative hazard rate transformed |
|  | Primary HF event date | 0.00 |  | Continuous; cumulative hazard rate transformed |
|  | Primary Stroke event date | 0.00 |  | Continuous; cumulative hazard rate transformed |

**Table S6. Baseline characteristics of total sample and complete case sample**

|  | **Complete case sample** | **Total sample** |
| --- | --- | --- |
| **n** | 6109 | 6334 |
| **Age, years, median [IQR])** | 66.85 [61.09, 70.54] | 66.85 [61.11, 70.59] |
| **Male sex, n (%)** | 2387 (39.1) | 2496 (39.4) |
| **White ethnicity, n (%)** | 5991 (98.1) | 6202 (97.9) |
| **Townsend deprivation index, median [IQR]** | -2.57 [-3.87, -0.43] | -2.56 [-3.85, -0.37] |
| **University education, n (%)** | 2650 (43.4) | 2715 (42.9) |
| **Smoking status, n (%)** |  |  |
| Never | 3351 (54.9) | 3446 (54.4) |
| Previous | 2392 (39.2) | 2501 (39.5) |
| Current | 366 (6.0) | 387 (6.1) |
| **Alcohol intake, grams / day, median [IQR]** |  |  |
| never | 1211(19.8) | 1271 (20.1) |
| moderate | 3297 (54.0) | 3411 (53.9) |
| excessive | 1601 (26.2) | 1652 (26.1) |
| **Diet score, n (%)** |  |  |
| 0 | 663 (10.9) | 688 (10.9) |
| 1 | 2258 (37.0) | 2342 (37.0) |
| 2 | 2207 (36.1) | 2288 (36.1) |
| 3 | 981 (16.1) | 1016 (16.0) |
| **Parental history of CVD, n (%)** | 3458 (56.6) | 3567 (56.3) |
| **Parental history of cancer, n (%)** | 2103 (34.4) | 2186 (34.5) |
| **Medication for cholesterol, n (%)** | 870(14.2) | 914 (14.4) |
| **Medication for blood pressure, n (%)** | 1182(19.3) | 1241 (19.6) |
| **Medication for diabetes, n (%)** | 34(0.6) | 34(0.5) |
| **Physical Measures and Biomarkers** |  |  |
| Systolic blood pressure, mmHg, median [IQR] | 138.00 [125.50, 151.00] | 138.00 [126.00, 151.00] |
| Diastolic blood pressure, mmHg, median [IQR] | 81.00 [74.50, 88.00] | 81.00 [74.50, 88.00] |
| Body mass index, kg/m^2^, median [IQR] | 26.02 [23.56, 29.07] | 26.06 [23.59, 29.11] |
| C reactive protein, mg/L, median [IQR] | 1.28 [0.66, 2.62] | 1.28 [0.66, 2.63] |
| eGFR, mL/min/1.73m^2^, median [IQR] | 95.61 [84.65, 101.18] | 95.61 [84.73, 101.18] |
| LDL direct, mmol/L, median [IQR] | 3.58 [3.01, 4.18] | 3.58 [3.01, 4.18] |
| Lipoprotein A, nmol/L, median [IQR] | 20.05 [9.45, 57.91] | 20.10 [9.40, 58.43] |
| HDL, mmol/L, median [IQR] | 1.45 [1.22, 1.75] | 1.45 [1.22, 1.75] |
| Triglycerides, mmol/L, median [IQR] | 1.47 [1.05, 2.10] | 1.48 [1.06, 2.10] |
| Glycated haemoglobin (HbA1c), mmol/mol, median [IQR] | 35.20 [33.00, 37.60] | 35.30 [33.00, 37.70] |
| **Interval between cancer diagnosis and wrist-worn completion, years, median [IQR]** | 6.92 [3.09, 11.95] | 6.90 [3.09, 11.90] |
| **Cancers, n (%)** |  |  |
| Non-obesity-related cancers, n (%) | 2708(44.3) | 2823 (44.6) |
| Obesity-related cancers ^a^, n (%) | 3401(55.7) | 3511 (55.4) |
| **Accelerometer data** |  | 137.64 (18.55) |
| Wear duration, days, median [IQR] | 6.91 [6.74, 7.00] | 6.91 [6.74, 7.00] |
| Sleep, h/d, median [IQR] | 8.45 [7.64, 9.28] | 8.44 [7.64, 9.28] |
| Moderate to vigorous physical activity, min/week, median [IQR] | 201.00[90.00, 358.80] | 199.20 [89.40, 357.60] |

| **Table S7. Calculation of pure alcohol intake in the UK Biobank cohort** | | | | | | |
| --- | --- | --- | --- | --- | --- | --- |
| Beverage categories | Red wine | Champagne / white wine | Beer / Cider | Spirits | Fortified Wine | Others |
| Units of alcohol* (10 ml of pure ethanol) | 1 glass = 175ml | 1 glass = 125ml | 1 pint = 568ml | 1 measure = 25ml | 1 glass = 75ml | 1 glass = 275ml |
|  | 2.3 Units | 1.5 Units | 2.3 Units | 1.0 Unit | 1.3 Units | 1.1 Unit |
| Alcohol by volume | 13.00% | 12.00% | 4.00% | 40.00% | 18.00% | 4.00% |
| Alcohol in grams | 18.4g | 12.0g | 18.4g | 8.0g | 10.4g | 8.8g |
| *The standardized alcohol units (1unit=8g) in different beverage categories were sourced from <https://www.drinkaware.co.uk/> | | | | | | |

| **Table S8. The scoring system of diet** | | | |
| --- | --- | --- | --- |
| Characteristics | Data field ID | Questions | Target Value |
| Fruit and Vegetables | 1289 | 1. On average how many heaped tablespoons of COOKED vegetables would you eat per DAY? | Sum ≥ 4.5 pieces or servings /day,  Yes (1) / No (0)  3 tablespoons of vegetables were considered one serving |
|  | 1299 | 2. On average how many heaped tablespoons of SALAD or RAW vegetables would you eat per DAY? |  |
|  | 1309 | 3. How many pieces of FRESH fruit would you eat daily? |  |
| Fish | 1329 | 4. How often do you eat oily fish? (e.g. sardines, salmon, mackerel, herring) | Sum ≥2 intake/week,  Yes (1) / No (0) |
|  | 1339 | 5. How often do you eat other types of fish? (e.g. cod, tinned tuna, haddock) |  |
| Processed Meat | 1349 | 6. How often do you eat processed meats (such as bacon, ham, sausages, meat pies, kebabs, burgers, chicken nuggets) | ≤ 2 times/week intake of processed meat  and ≤ 5 times/week of red meat intake  , Yes (1) / No (0) |
| Red Meat | 1359 | 7. How often do you eat chicken, turkey or other poultry? (Do not count processed meats) |  |
|  | 1369 | 8. How often do you eat beef? (Do not count processed meats) |  |
|  | 1379 | 9. How often do you eat lamb/mutton? (Do not count processed meats) |  |
|  | 1389 | 10. How often do you eat pork? (Do not count processed meats such as bacon or ham) |  |

**Table S9. Associations of Moderate to vigorous physical activity with incident cardiovascular events in the Cause-Specific Hazard Model and Fine-gray Model**

|  | | **Cause-Specific Hazard Model** | | | | |  | **Fine-gray Model** | |
| --- | --- | --- | --- | --- | --- | --- | --- | --- | --- |
|  | | **HR (95% CI)** | | | **P value** | |  | **HR (95% CI)** | **P value** |
| **Cardiovascular diseases** | |  | | |  | |  |  |  |
| **MVPA (min/week)** | |  | | |  | |  |  |  |
| **0≤MVPA<75** | | Ref | | |  | |  | Ref |  |
| **75≤MVPA<150** | | 0.86 (0.67-1.12) | | | 0.263 | |  | 0.89 (0.69-1.15) | 0.37 |
| **150≤MVPA<300** | | **0.77 (0.61-0.97)** | | | **0.026** | |  | 0.79 (0.63-1.00) | 0.052 |
| **MVPA≥300** | | **0.63 (0.49-0.80)** | | | **<0.001** | |  | **0.66 (0.52-0.84)** | **<0.001** |
| **Coronary artery diseases** | |  | | |  | |  |  |  |
| **MVPA (min/week)** | |  | | |  | |  |  |  |
| **0≤MVPA<75** | | Ref | | |  | |  | Ref |  |
| **75≤MVPA<150** | | 0.85 (0.62-1.17) | | | 0.311 | |  | 0.87 (0.63-1.20) | 0.39 |
| **150≤MVPA<300** | | 0.78 (0.58-1.04) | | | 0.09 | |  | 0.81 (0.61-1.08) | 0.15 |
| **MVPA≥300** | | **0.68 (0.51-0.91)** | | | **0.011** | |  | **0.72 (0.54-0.94)** | **0.024** |
| **Heart Failure** |  | |  |  | |  |  |  |  |
| **MVPA (min/week)** | |  | | |  | |  |  |  |
| **0≤MVPA<75** | | Ref | | |  | |  | Ref |  |
| **75≤MVPA<150** | | 1.06 (0.66-1.69) | | | 0.817 | |  | 1.10 (0.69-1.76) | 0.70 |
| **150≤MVPA<300** | | 0.80 (0.51-1.25) | | | 0.334 | |  | 0.83 (0.53-1.31) | 0.43 |
| **MVPA≥300** | | 0.66 (0.42-1.06) | | | 0.084 | |  | 0.71 (0.44-1.14) | 0.15 |
| **Stroke** | |  | | |  | |  |  |  |
| **MVPA (min/week)** | |  | | |  | |  |  |  |
| **0≤MVPA<75** | | Ref | | |  | |  | Ref |  |
| **75≤MVPA<150** | | 0.91 (0.51-1.60) | | | 0.735 | |  | 0.94(0.53-1.65) | 0.82 |
| **150≤MVPA<300** | | 0.77 (0.46-1.31) | | | 0.339 | |  | 0.81(0.47-1.38) | 0.44 |
| **MVPA≥300** | | 0.72 (0.42-1.23) | | | 0.227 | |  | 0.76(0.45-1.29) | 0.32 |

The models use the completion of the accelerometer worn as the timescale and covariates from Model 2.

**Table S10. Interaction Effects of Moderate to Vigorous Physical Activity and Risk Factors on the incidence of cardiovascular diseases**

|  | Multiplicative interaction HR (95%CI); P | Additive interaction | | |
| --- | --- | --- | --- | --- |
|  |  | RERI (95%CI) | AP (95%CI) | S (95%CI) |
| MVPA above WHO recommendation (vs MVPA below WHO recommendation) & older age of the cancer diagnosis (≥60 vs ＜60 years) | 1.12（0.79, 1.58）; 0.53 | 0.53(-0.06, 1.12) | 0.19(-0.01, 0.40) | 1.46 (0.92, 2.34) |
| MVPA above WHO recommendation (vs MVPA below WHO recommendation) & Male | 0.94(0.66, 1.33); 0.72 | 0.15(-0.43, 0.75) | 0.07(-0.18, 0.31) | 1.13(0.70, 1.83) |
| MVPA above WHO recommendation (vs MVPA below WHO recommendation) & no University degree | 1.09(0.76,1.55); 0.65 | 0.14(-0.29, 0.56) | 0.09(-0.20, 0.38) | 1.39(0.40, 4.83) |
| MVPA above WHO recommendation (vs MVPA below WHO recommendation) & smoking | 1.04(0.74, 1.46); 0.83 | 0.20(-0.28, 0.69) | 0.10(-0.14, 0.35) | 1.28(0.67, 2.42) |
| MVPA above WHO recommendation (vs MVPA below WHO recommendation) & alcohol(≥median vs <median) | 0.81(0.52, 1.26); 0.35 | -0.27(-0.89, 0.34) | -0.19(-0.60, 0.21) | 0.60(0.27, 1.34) |
| MVPA above WHO recommendation (vs MVPA below WHO recommendation) & diet (2,3 vs 0,1) | 0.81(0.58, 1.14); 0.22 | -0.27(-0.71,0.17) | -0.21(-0.57, 0.14) | 0.49(0.17, 1.43) |
| MVPA above WHO recommendation (vs MVPA below WHO recommendation) & sleep duration (≥7h vs <7h) | 0.96(0.57, 1.61); 0.88 | -0.03(-0.67,0.62) | -0.02(-0.49,0.45) | 0.94(0.19, 4.58) |
| MVPA above WHO recommendation (vs MVPA below WHO recommendation) & hypertension | 0.89(0.64,1.26); 0.49 | 0.01(-0.53, 0.55) | 0.01(-0.26, 0.27) | 1.01(0.59, 1.72) |
| MVPA above WHO recommendation (vs MVPA below WHO recommendation) & Diabetes | 1.90(0.97,3.73); 0.06 | **1.60(0.33, 2.88)** | **0.50(0.21, 0.79)** | 3.70(0.91, 15.06) |
| MVPA above WHO recommendation (vs MVPA below WHO recommendation) & BMI (≥25 vs <25Kg/m2) | 1.06(0.71, 1.56); 0.78 | 0.14(-0.15,0.44) | 0.09(-0.13,0.30) | 1.29(0.53, 3.16) |
| MVPA above WHO recommendation (vs MVPA below WHO recommendation) & LDL (≥3.4 vs <3.4mmol/l) | 0.79(0.55,1.12); 0.19 | -0.37(-1.03, 0.29) | -0.23(-0.56, 0.11) | 0.63(0.41,0.98) |
| MVPA above WHO recommendation (vs MVPA below WHO recommendation) & TG (≥1.7 vs <1.7mmol/l) | 0.76(0.53,1.08); 0.12 | -0.38(-1.03,0.27) | -0.21(-0.51,0.09) | 0.68(0.47,1.01) |

Cox proportional hazard regression was used to examine the associations of MVPA timing and sex with mortality risk, which were adjusted for age, sex, education, Townsend Deprivation Index, ethnicity(white/non-white), medication (cholesterol, blood pressure, or diabetes), smoking status, alcohol consumption, diet score, sleep duration, parental history of CVD or cancer, and years since first cancer diagnosis. Multiplicative and additive interaction analyses were performed to examine the interaction effects. AP: attributable proportion due to interaction; CI: confidence interval; HR: hazard ratio; MVPA: moderate to vigorous physical activity; RERI: relative excess risk due to interaction; S: synergy index.

**Table S11. Adjusted hazard ratio (95% CI) for cardiovascular diseases associated with MVPA in subgroup analysis**

| **Subgroup Analysis** | | **Cardiovascular diseases ^a^** | | | **P for trend** |
| --- | --- | --- | --- | --- | --- |
|  |  | **MVPA (75-150)** | **MVPA (150-300)** | **MVPA (≥300)** |  |
| Age of the cancer diagnosis  (years) | <60 | 0.62 (0.41-0.96) | 0.71 (0.49-1.03) | 0.61 (0.42-0.88) | 0.03 |
|  | ≥60 | 1.05 (0.76-1.45) | 0.80 (0.59-1.09) | 0.63 (0.46-0.88) | 0.001 |
| Sex | Women | 0.86 (0.61-1.22) | 0.82 (0.59-1.14) | 0.54 (0.36-0.81) | 0.003 |
|  | Men | 0.87 (0.59-1.28) | 0.73 (0.52-1.02) | 0.66 (0.48-0.91) | 0.02 |
| University Education | No | 0.85 (0.62-1.16) | 0.75 (0.56-1.00) | 0.54 (0.40-0.75) | <0.001 |
|  | Yes | 0.87 (0.55-1.38) | 0.79 (0.53-1.18) | 0.76 (0.51-1.14) | 0.24 |
| Smoking Status | Never | 1.44 (0.96-2.14) | 1.00 (0.68-1.48) | 0.81 (0.54-1.22) | 0.045 |
|  | Current  or Previous | 0.59 (0.41-0.85) | 0.67 (0.50-0.91) | 0.56 (0.41-0.76) | 0.002 |
| Alcohol intake | ＜median | 0.83 (0.49-1.42) | 0.67 (0.39-1.14) | 0.49 (0.27-0.91) | 0.02 |
|  | ≥median | 0.87 (0.65-1.17) | 0.79 (0.61-1.03) | 0.66 (0.50-0.86) | 0.003 |
| Diet score | 0,1 | 0.78 (0.55-1.12) | 0.62 (0.44-0.86) | 0.67 (0.48-0.93) | 0.03 |
|  | 2,3 | 0.97 (0.67-1.40) | 0.95 (0.68-1.34) | 0.61 (0.43-0.88) | 0.003 |
| Sleep duration  (h/d) | <7 | 1.16 (0.55-2.45) | 0.77 (0.39-1.53) | 0.98 (0.49-1.98) | 0.84 |
|  | ≥7 | 0.83 (0.63-1.10) | 0.76 (0.59-0.97) | 0.59 (0.46-0.77) | <0.001 |
| Hypertension | No | 0.89 (0.62-1.27) | 0.73 (0.53-1.01) | 0.60 (0.43-0.84) | 0.002 |
|  | Yes | 0.82 (0.57-1.19) | 0.82 (0.59-1.15) | 0.67 (0.47-0.97) | 0.04 |
| Diabetes | No | 0.92 (0.70-1.21) | 0.84 (0.66-1.08) | 0.71 (0.54-0.91) | 0.006 |
|  | Yes | 0.76 (0.34-1.70) | 0.43 (0.18-1.02) | 0.21 (0.06-0.72) | 0.014 |
| BMI(Kg/m^2^) | <25 | 0.63 (0.36-1.10) | 0.73 (0.46-1.17) | 0.56 (0.34-0.90) | 0.04 |
|  | ≥25 | 0.99 (0.74-1.32) | 0.80 (0.61-1.05) | 0.70 (0.52-0.93) | 0.01 |
| LDL-C (mmol/l) | <3.4 | 0.80 (0.55-1.19) | 0.66 (0.46-0.95) | 0.55 (0.38-0.81) | 0.003 |
|  | ≥3.4 | 1.05 (0.73-1.52) | 0.95 (0.67-1.33) | 0.76 (0.54-1.08) | 0.06 |
| Triglycerides  (mmol/l) | <1.7 | 0.89 (0.62-1.27) | 0.63 (0.45-0.89) | 0.55 (0.39-0.77) | 0.001 |
|  | ≥1.7 | 1.01 (0.68-1.49) | 1.03 (0.72-1.48) | 0.82 (0.56-1.21) | 0.28 |

a.With the first MVPA group (0-75 min/week) as reference. The adjusted hazard ratio was estimated using a Cox proportional hazards model, with the completion of the accelerometer worn as the timescale and covariates from Model 2.

| Table S12. STROBE Statement—checklist of items that should be included in reports of observational studies | | | |
| --- | --- | --- | --- |
|  | **Item** | **Recommendation** | **Page** |
| Title and abstract | 1 | (a) Indicate the study’s design with a commonly used term in the title or the abstract | 3 |
|  |  | (b) Provide in the abstract an informative and balanced summary of what was done and what was found | 3,4 |
| Introduction | | | |
| Background/rationale | 2 | Explain the scientific background and rationale for the investigation being reported | 6,7 |
| Objectives | 3 | State specific objectives, including any prespecified hypotheses | 7 |
| Methods | | | |
| Study design | 4 | Present key elements of study design early in the paper | 7-10 |
| Setting | 5 | Describe the setting, locations, and relevant dates, including periods of recruitment, exposure, follow-up, and data collection | 7-10 |
| Participants | 6 | (a) Cohort study—Give the eligibility criteria, and the sources and methods of selection of participants. Describe methods of follow-up | 7-8 |
|  |  | (b) Cohort study—For matched studies, give matching criteria and number of exposed and unexposed | NA |
| Variables | 7 | Clearly define all outcomes, exposures, predictors, potential confounders, and effect modifiers. Give diagnostic criteria, if applicable | 7-8 |
| Data sources/ measurement | 8 | For each variable of interest, give sources of data and details of methods of assessment (measurement). Describe comparability of assessment methods if there is more than one group. | 7-8 |
| Bias | 9 | Describe any efforts to address potential sources of bias | 17-18 |
| Study size | 10 | Explain how the study size was arrived at | 7-8 |
| Quantitative variables | 11 | Explain how quantitative variables were handled in the analyses. If applicable, describe which groupings were chosen and why | 8-9 |
| Statistical methods | 12 | (a) Describe all statistical methods, including those used to control for confounding | 9-10 |
|  |  | (b) Describe any methods used to examine subgroups and interactions | 10 |
|  |  | (c) Explain how missing data were addressed | 9 |
|  |  | (d) Cohort study—If applicable, explain how loss to follow-up was addressed | 9 |
|  |  | (e) Describe any sensitivity analyses | 9-10 |
| Results | | | |
| Participants | 13 | (a) Report numbers of individuals at each stage of study—eg numbers potentially eligible, examined for eligibility, confirmed eligible, included in the study, completing follow-up, and analyzed | Figure 1 & S1  11 |
|  |  | (b) Give reasons for non-participation at each stage |  |
|  |  | (c) Consider use of a flow diagram |  |
| Descriptive data | 14 | (a) Give characteristics of study participants (eg. Demographic, clinical, social) and information on exposures and potential confounders | Table 1 |
|  |  | (b) Indicate number of participants with missing data for each variable of interest | Figure S1 |
|  |  | (c) Cohort study—Summarize follow-up time (eg, average and total amount) | 11 |
| Outcome data | 15 | Cohort study—Report numbers of outcome events or summary measures over time | 11, Figure 1 & S1 |
| Main results | 16 | (a) Give unadjusted estimates and, if applicable, confounder- adjusted estimates and their precision (eg, 95% confidence interval). Make clear which confounders were adjusted for and why they were included | 13,14,  Figure 2和S2 |
|  |  | (b) Report category boundaries when continuous variables were categorized | Figure 2 |
|  |  | (c) If relevant, consider translating estimates of relative risk into absolute risk for a meaningful time period | Table 2 |
| Other analyses | 17 | Report other analyses done—eg analyses of subgroups and interactions, and sensitivity analyses | 13-14 |
| Discussion | | | |
| Key results | 18 | Summarize key results with reference to study objectives | 14-16 |
| Limitations | 19 | Discuss limitations of the study, taking into account sources of potential bias or imprecision. Discuss both direction and magnitude of any potential bias | 17-18 |
| Interpretation | 20 | Give a cautious overall interpretation of results considering objectives, limitations, multiplicity of analyses, results from similar studies, and other relevant evidence | 15-17 |
| Generalizability | 21 | Discuss the generalizability (external validity) of the study results | 15-16 |
| Other information | | | |
| Funding | 22 | Give the source of funding and the role of the funders for the present study and, if applicable, for the original study on which the present article is based | 19 |

**Figure S1. Selection of the study population.**


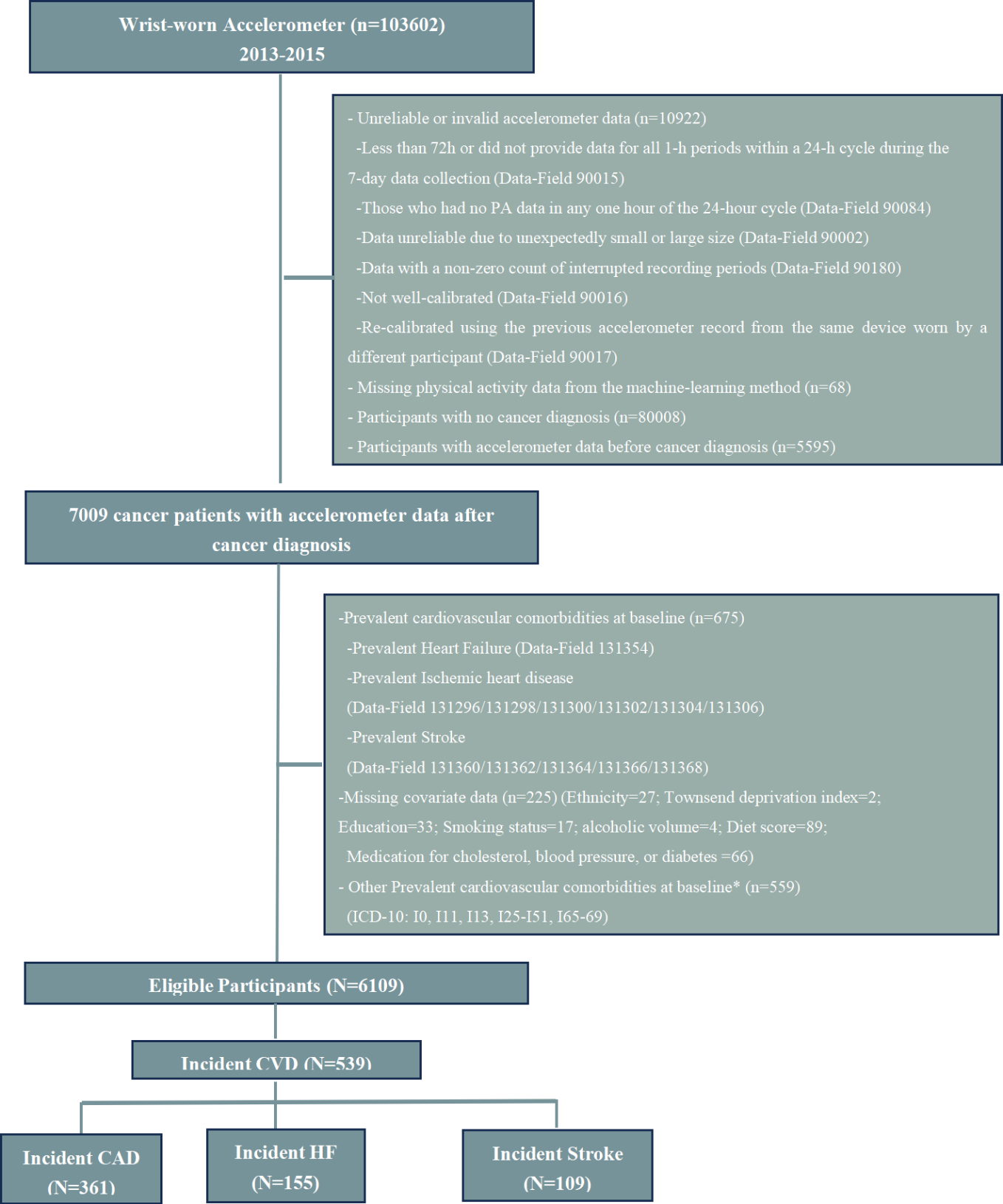


*The criterion for exclusion applies only in the sensitivity analysis. CVD, cardiovascular disease; CAD, coronary artery disease; HF, Heart failure.

**Figure S2. Directed acyclic graph to guide covariate selection**


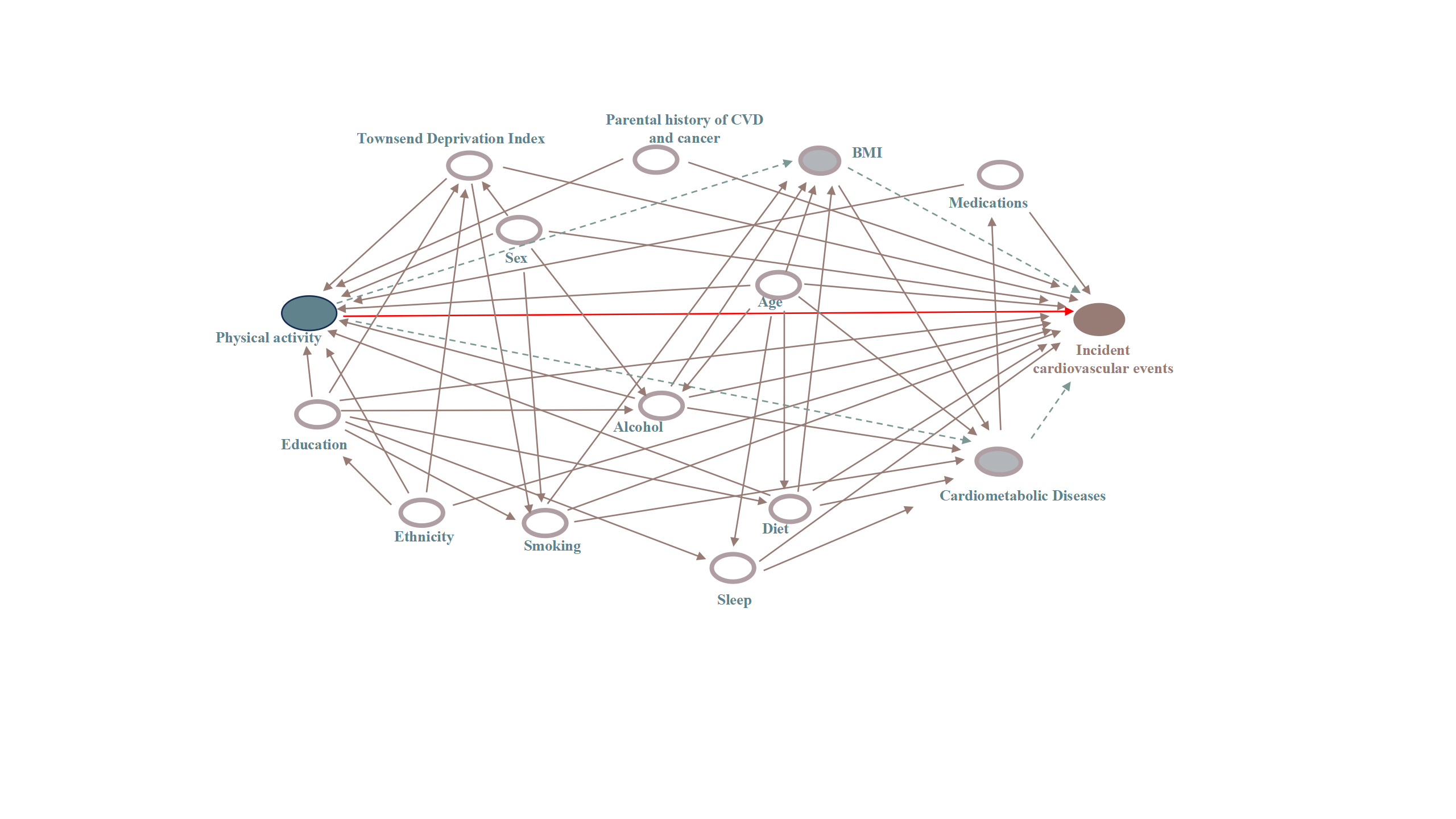


Green circle: exposure; Brown circle: outcome; Gray circle: mediators;

Cardiometabolic diseases: obesity, diabetes, hypertension, and dyslipidemia
Each clinical factor included in the model is depicted as a named node. BMI and Cardiometabolic diseases are potential mediators between physical activity and cardiovascular diseases and are displayed in gray.

**Figure S3. Dose-response relationship between moderate-to-vigorous intensity physical activity volume and risk of incident cardiovascular events.**


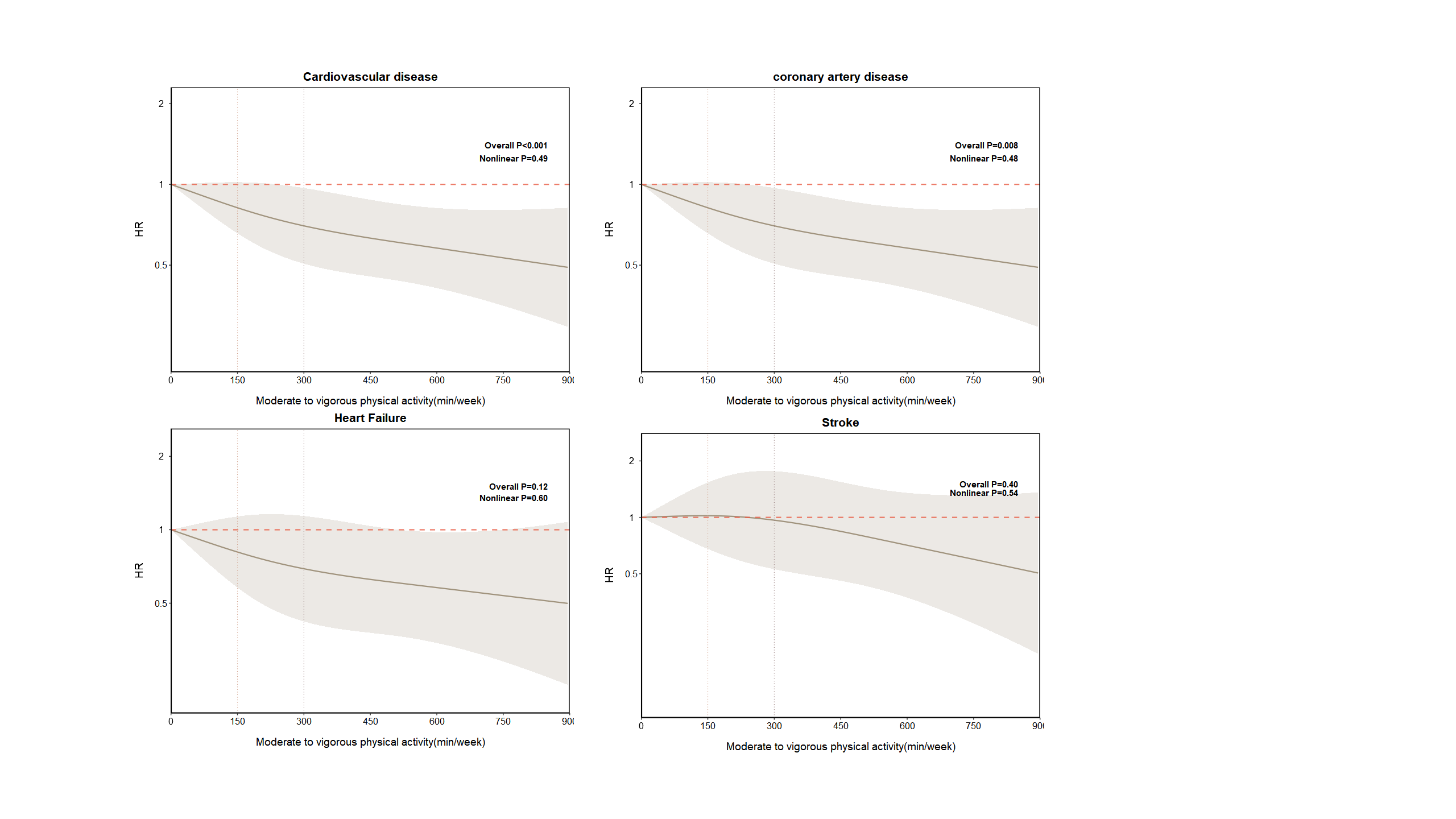


The relative risk of cardiovascular diseases by moderate-to-vigorous intensity physical activity volume. The adjusted hazard ratio was estimated using the Cox proportional hazards model, with time since the completion of the accelerometer worn as the timescale. Adjustments were made for age, sex, education, Townsend Deprivation Index, ethnicity(white/non-white), medication (cholesterol, blood pressure, or diabetes), smoking status, alcohol consumption, diet score, time spent in sleep, parental history of CVD or cancer, and years since first cancer diagnosis. The solid line represents the adjusted hazard ratio, with the ribbon indicating the 95% confidence interval. The gray area denotes the population proportion (units: 30 min/week). CI: confidence interval.

**Figure S4. Adjusted survival curves for incident cardiovascular events risk by guideline-recommended Moderate to vigorous physical activity.**


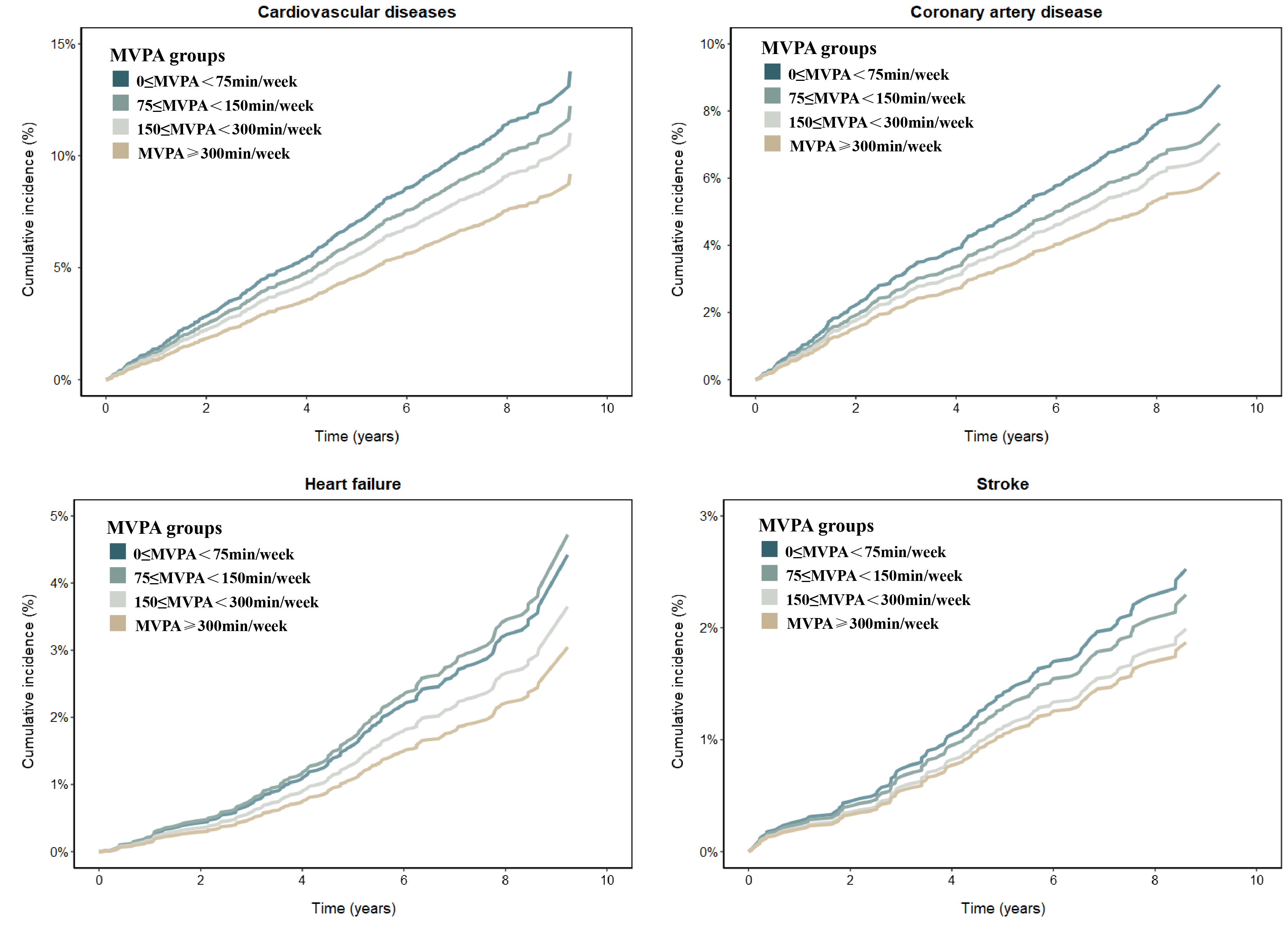


Adjusted survival curves for incident cardiovascular events risk by guideline-recommended Moderate to vigorous physical activity. The adjusted hazard ratio was estimated using the Cox proportional hazards model, with follow-up years as the timescale. Adjustments were made for age, sex, education, Townsend Deprivation Index, ethnicity(white/non-white), medication (cholesterol, blood pressure, or diabetes), smoking status, alcohol consumption, diet score, time spent in sleep, parental history of CVD or cancer, and years since first cancer diagnosis.

**Figure S5. Estimated cumulative risk of incident cardiovascular events since the diagnosis of cancer.**

| **Age of the cancer diagnosis**  **(years)** | **MVPA (min/wk)** | **Absolute 3-year risk of CVD (%)** | | **Absolute 5-year risk of CVD (%)** | |
| --- | --- | --- | --- | --- | --- |
|  |  | **Women** | **Men** | **Women** | **Men** |
| **40-59** | **0≤MVPA＜75** | **1.6** | **8.7** | **4.1** | **9.6** |
|  | **75≤MVPA＜150** | **1.0** | **6.6** | **1.7** | **6.6** |
|  | **MVPA ≥150** | **1.5** | **4.4** | **2.2** | **6.8** |
|  | | | | | |
| **60-79** | **0≤MVPA＜75** | **5.1** | **7.4** | **7.3** | **13.5** |
|  | **75≤MVPA＜150** | **4.2** | **8.5** | **7.2** | **12.9** |
|  | **MVPA ≥150** | **2.7** | **4.8** | **4.1** | **8.4** |

Estimated cumulative risk of incident cardiovascular events since the wrist-worn accelerometer completion. We used the Fine and Gray competing risks regression model to estimate the 3- and 5-year cumulative risk of cardiovascular diseases stratified by sex and age of cancer diagnosis, using years of follow-up as the timescale.

**Figure S6. Dose-response relationship between moderate-to-vigorous intensity physical activity volume and risk of incident cardiovascular events among the obesity and non-obesity-related cancer survivors.**


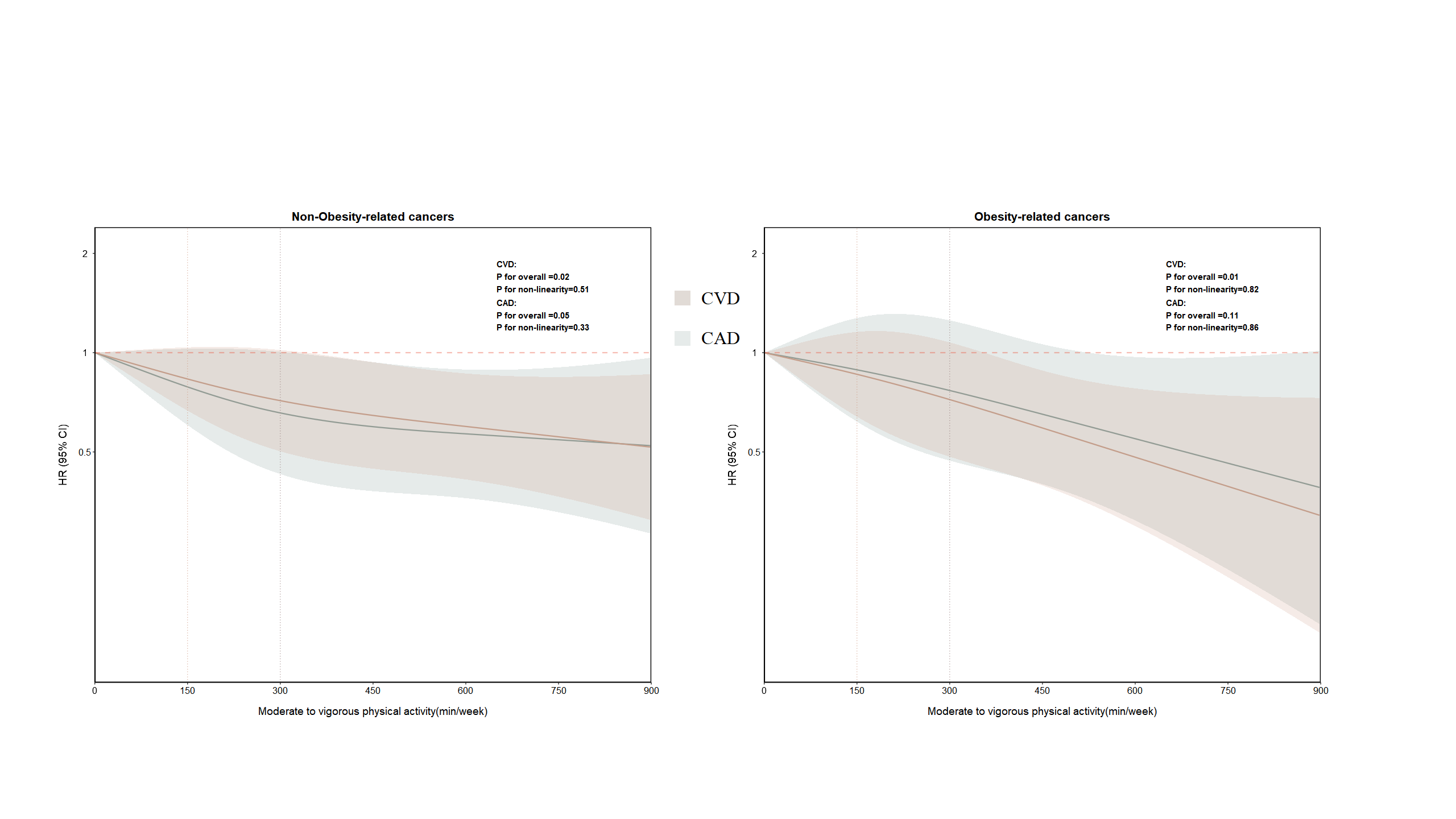


The adjusted hazard ratio was estimated using the Cox proportional hazards model, with time since the completion of the accelerometer worn as the timescale. Adjustments were made for age, sex, education, Townsend Deprivation Index, ethnicity(white/non-white), medication (cholesterol, blood pressure, or diabetes), smoking status, alcohol consumption, diet score, time spent in sleep, parental history of CVD or cancer, and years since first cancer diagnosis. The solid line represents the adjusted hazard ratio, with the ribbon indicating the 95% confidence interval. The gray area denotes the population proportion (units: 30 min/week). CI: confidence interval.

**Figure S7. Adjusted hazard ratio for incident cardiovascular events by guideline-recommended Moderate to vigorous physical activity after additionally adjusting the body mass index (BMI).**


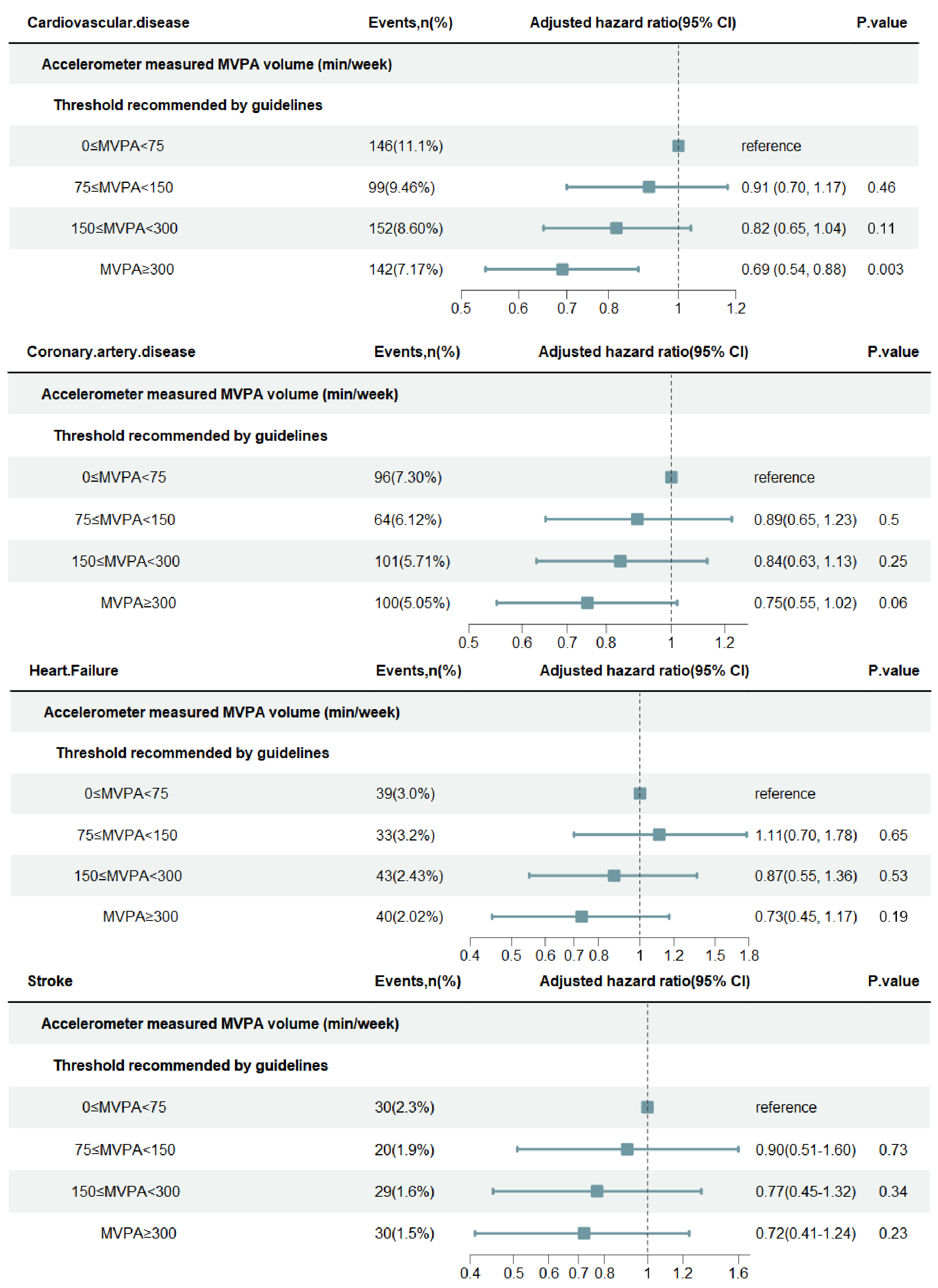


The adjusted hazard ratio was estimated using the Cox proportional hazards model, with time since the completion of the accelerometer worn as the timescale and covariate from Model 2.

**Figure S8. Adjusted hazard ratio for incident cardiovascular events by guideline-recommended Moderate to vigorous physical activity additionally excluding participants with other cardiovascular diseases.**


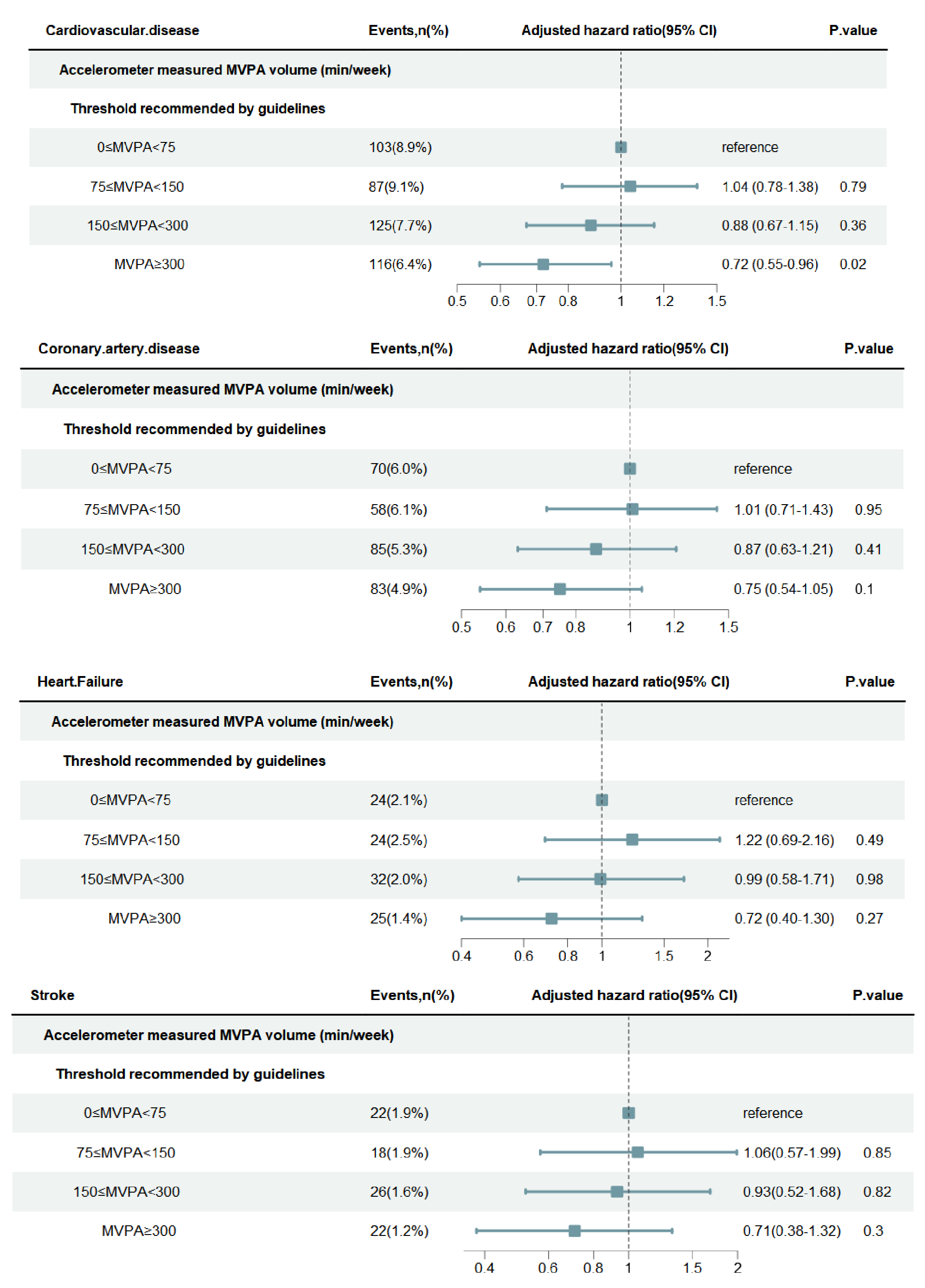


The adjusted hazard ratio was estimated using the Cox proportional hazards model, with time since the completion of the accelerometer worn as the timescale and covariate from Model 2.

**Figure S9. Adjusted hazard ratio for incident cardiovascular events by guideline-recommended Moderate to vigorous physical activity excluding participants with an event in the initial 2 years of follow-up.**


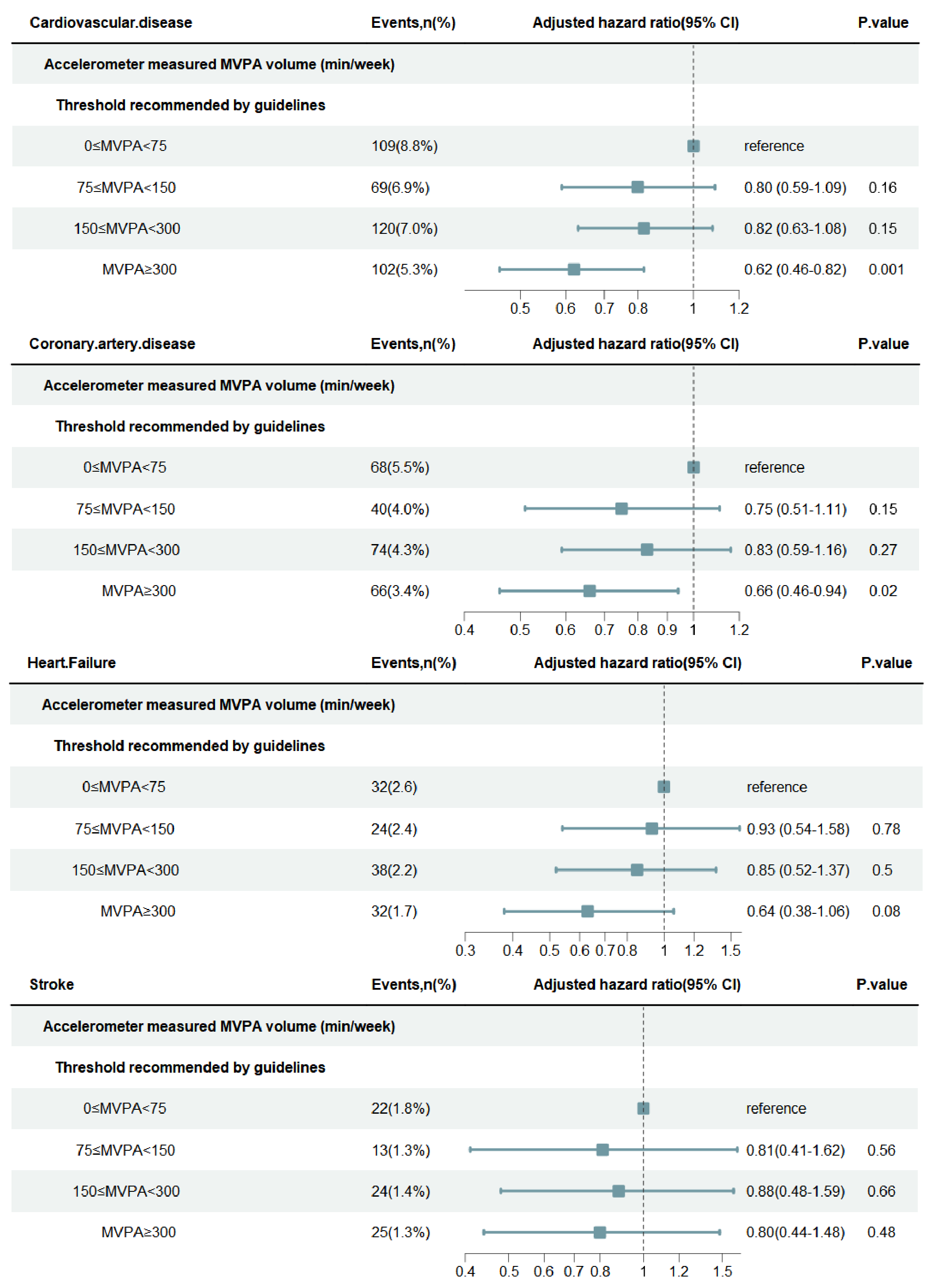


The adjusted hazard ratio was estimated using the Cox proportional hazards model, with time since the completion of the accelerometer worn as the timescale and covariate from Model 2.

**Figure S10. Adjusted hazard ratio for incident cardiovascular events by different Moderate to vigorous physical activity groups in the total sample. (multiple imputation of covariates)**

**
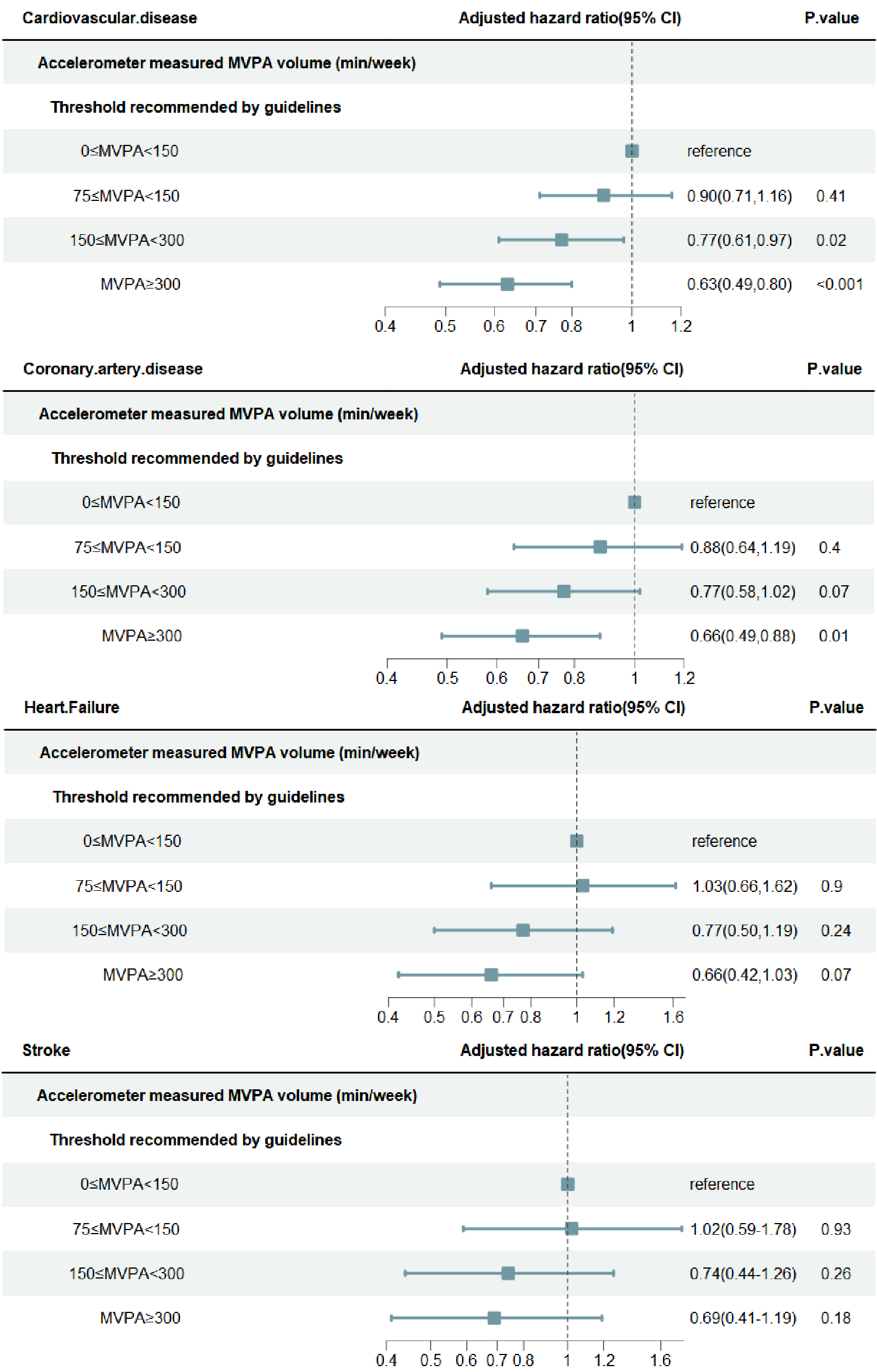
**

The adjusted hazard ratio was estimated using the Cox proportional hazards model, with time since the completion of the accelerometer worn as the timescale and covariate from Model 2.

**Figure S11.** **Adjusted hazard ratio for incident cardiovascular events by different Moderate to vigorous physical activity groups.**


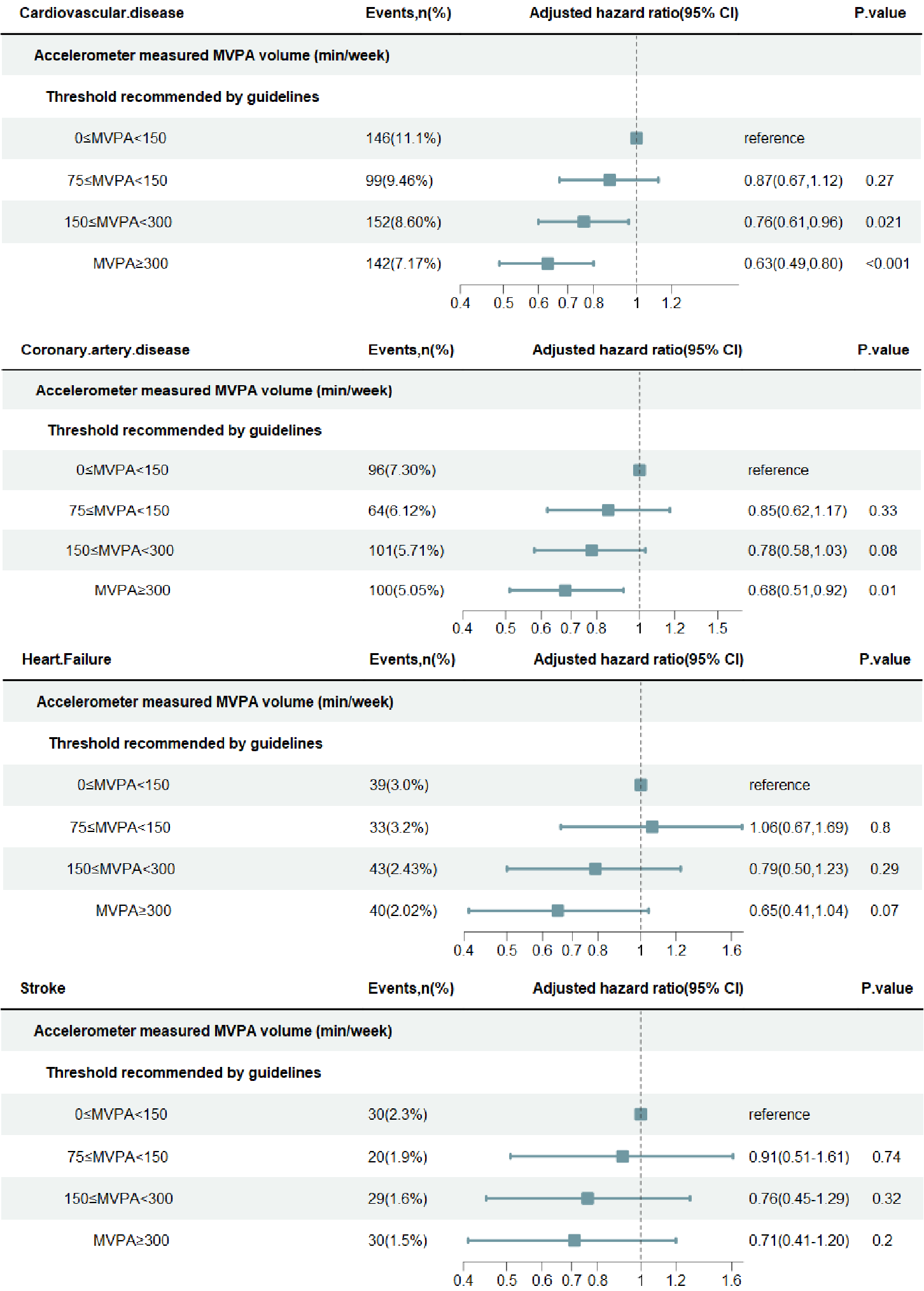


The adjusted hazard ratio was estimated using the Cox proportional hazards model, with time since the completion of the accelerometer worn as the timescale and covariates from the interview closest to the accelerometer assessment.

**Figure S12. Adjusted hazard ratio for incident cardiovascular events by guideline-recommended Moderate to vigorous physical activity.**


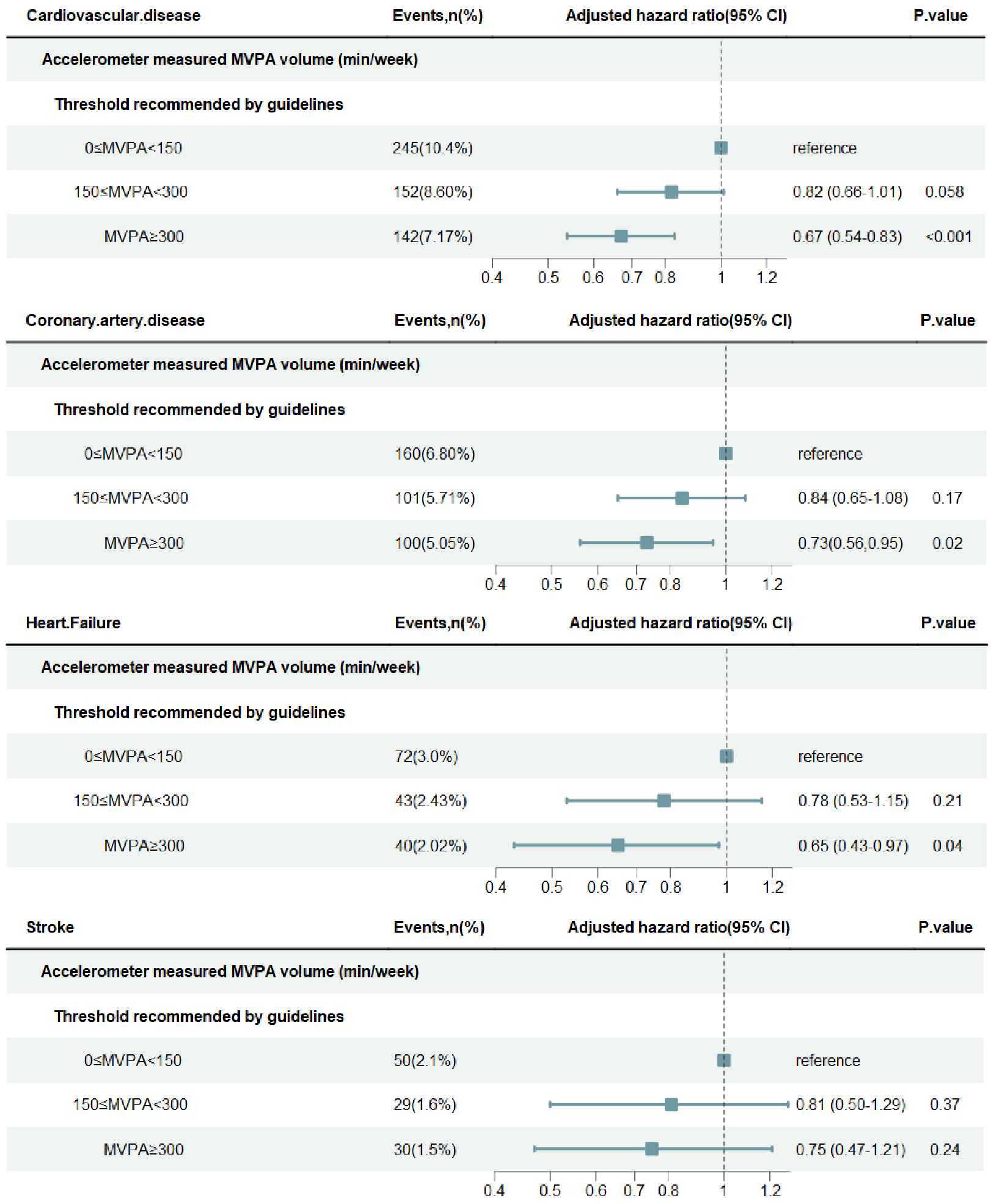


The adjusted hazard ratio was estimated using the Cox proportional hazards model, with time since the completion of the accelerometer worn as the timescale and covariate from Model 2.

**Reference**

32. De Angelis R, Sant M, Coleman MP, et al. Cancer survival in Europe 1999-2007 by country and age: results of EUROCARE--5-a population-based study. Lancet Oncol. 2014;15(1):23-34. doi:10.1016/S1470-2045(13)70546-1

33. Araghi M, Arnold M, Rutherford MJ, et al. Colon and rectal cancer survival in seven high-income countries 2010-2014: variation by age and stage at diagnosis (the ICBP SURVMARK-2 project). Gut. 2021;70(1):114-126. doi:10.1136/gutjnl-2020-320625

34. Keegan THM, Abrahão R, Alvarez EM. Survival Trends Among Adolescents and Young Adults Diagnosed with Cancer in the United States: Comparisons with Children and Older Adults. J Clin Oncol. 2024;42(6):630-641. doi:10.1200/JCO.23.01367

35. Havranek EP, Mujahid MS, Barr DA, et al. Social Determinants of Risk and Outcomes for Cardiovascular Disease: A Scientific Statement from the American Heart Association. Circulation. 2015;132(9):873-898. doi:10.1161/CIR.0000000000000228

36. Yang W, Giovannucci EL, Hankinson SE, et al. Endogenous sex hormones and colorectal cancer survival among men and women. Int J Cancer. 2020;147(4):920-930. doi:10.1002/ijc.32844

37. Ellison LF. Differences in cancer survival in Canada by sex. Health Rep. 2016;27(4):19-27.

38. Siegel DA, O'Neil ME, Richards TB, Dowling NF, Weir HK. Prostate Cancer Incidence and Survival, by Stage and Race/Ethnicity - United States, 2001-2017. MMWR Morb Mortal Wkly Rep. 2020;69(41):1473-1480. 2020 Oct 16. doi:10.15585/mmwr.mm6941a1

39. Miller JW, Smith JL, Ryerson AB, Tucker TC, Allemani C. Disparities in breast cancer survival in the United States (2001-2009): Findings from the CONCORD-2 study. Cancer. 2017;123 Suppl 24(Suppl 24):5100-5118. doi:10.1002/cncr.30988

40. White A, Joseph D, Rim SH, Johnson CJ, Coleman MP, Allemani C. Colon cancer survival in the United States by race and stage (2001-2009): Findings from the CONCORD-2 study. Cancer. 2017;123 Suppl 24(Suppl 24):5014-5036. doi:10.1002/cncr.31076

41. Zeng H, Chen W, Zheng R, et al. Changing cancer survival in China during 2003-15: a pooled analysis of 17 population-based cancer registries. Lancet Glob Health. 2018;6(5): e555-e567. doi:10.1016/S2214-109X (18)30127-X

42. Arnold M, Rutherford MJ, Bardot A, et al. Progress in cancer survival, mortality, and incidence in seven high-income countries 1995-2014 (ICBP SURVMARK-2): a population-based study. Lancet Oncol. 2019;20(11):1493-1505. doi:10.1016/S1470-2045(19)30456-5

43. Aarts MJ, Kamphuis CB, Louwman MJ, Coebergh JW, Mackenbach JP, van Lenthe FJ. Educational inequalities in cancer survival: a role for comorbidities and health behaviors? J Epidemiol Community Health. 2013;67(4):365-373. doi:10.1136/jech-2012-201404

44. Hussain SK, Lenner P, Sundquist J, Hemminki K. Influence of education level on cancer survival in Sweden. Ann Oncol. 2008;19(1):156-162. doi:10.1093/annonc/mdm413

45. Gillison ML, Zhang Q, Jordan R, et al. Tobacco smoking and increased risk of death and progression for patients with p16-positive and p16-negative oropharyngeal cancer. J Clin Oncol. 2012;30(17):2102-2111. doi:10.1200/JCO.2011.38.4099

46. Bérubé S, Lemieux J, Moore L, Maunsell E, Brisson J. Smoking at time of diagnosis and breast cancer-specific survival: new findings and systematic review with meta-analysis. Breast Cancer Res. 2014;16(2):R42. 2014 Apr 19. doi:10.1186/bcr3646

47. Yuan C, Morales-Oyarvide V, Babic A, et al. Cigarette Smoking and Pancreatic Cancer Survival. J Clin Oncol. 2017;35(16):1822-1828. doi:10.1200/JCO.2016.71.2026

48. Wang T, Townsend MK, Simmons V, Terry KL, Matulonis UA, Tworoger SS. Prediagnosis and postdiagnosis smoking and survival following diagnosis with ovarian cancer. Int J Cancer. 2020;147(3):736-746. doi:10.1002/ijc.32773

49. Kenfield SA, Stampfer MJ, Chan JM, Giovannucci E. Smoking and prostate cancer survival and recurrence. JAMA. 2011;305(24):2548-2555. doi:10.1001/jama.2011.879

50. Zhu Y, Yang SR, Wang PP, et al. Influence of pre-diagnostic cigarette smoking on colorectal cancer survival: overall and by tumor molecular phenotype. Br J Cancer. 2014;110(5):1359-1366. doi:10.1038/bjc.2014.6

51. Bian Z, Zhang R, Yuan S, et al. Healthy lifestyle and cancer survival: A multinational cohort study. Int J Cancer. 2024;154(10):1709-1718. doi:10.1002/ijc.34846

52. Kim Y, Je Y, Giovannucci EL. Association between Alcohol Consumption and Survival in Colorectal Cancer: A Meta-analysis. Cancer Epidemiol Biomarkers Prev. 2019;28(11):1891-1901. doi:10.1158/1055-9965.

53. Schwedhelm C, Boeing H, Hoffmann G, Aleksandrova K, Schwingshackl L. Effect of diet on mortality and cancer recurrence among cancer survivors: a systematic review and meta-analysis of cohort studies. Nutr Rev. 2016;74(12):737-748. doi:10.1093/nutrit/nuw045

54. Jochems SHJ, Van Osch FHM, Bryan RT, et al. Impact of dietary patterns and the main food groups on mortality and recurrence in cancer survivors: a systematic review of current epidemiological literature. BMJ Open. 2018;8(2):e014530. 2018 Feb 19. doi:10.1136/bmjopen-2016-014530

55. Hurtado-Barroso S, Trius-Soler M, Lamuela-Raventós RM, Zamora-Ros R. Vegetable and Fruit Consumption and Prognosis Among Cancer Survivors: A Systematic Review and Meta-Analysis of Cohort Studies. Adv Nutr. 2020;11(6):1569-1582. doi:10.1093/advances/nmaa082

56. Nair NM, Vaughn CB, Ochs-Balcom HM, Nie J, Trevisan M, Freudenheim JL. Sleep duration and mortality among breast cancer survivors in the Western New York Exposures and Breast Cancer (WEB) Study. Cancer Causes Control. 2024;35(1):103-109. doi:10.1007/s10552-023-01774-z

57. Lloyd-Jones DM, Allen NB, Anderson CAM, et al. Life's Essential 8: Updating and Enhancing the American Heart Association's Construct of Cardiovascular Health: A Presidential Advisory from the American Heart Association. Circulation. 2022;146(5): e18-e43. doi:10.1161/CIR.0000000000001078

58. Petrelli F, Ghidini A, Cabiddu M, et al. Effects of hypertension on cancer survival: A meta-analysis. Eur J Clin Invest. 2021;51(6):e13493. doi:10.1111/eci.13493

59. Rapsomaniki E, Timmis A, George J, et al. Blood pressure and incidence of twelve cardiovascular diseases: lifetime risks, healthy life-years lost, and age-specific associations in 1·25 million people. Lancet. 2014;383(9932):1899-1911. doi:10.1016/S0140-6736(14)60685-1

60. Cespedes Feliciano EM, Kroenke CH, Meyerhardt JA, et al. Metabolic Dysfunction, Obesity, and Survival Among Patients with Early-Stage Colorectal Cancer. J Clin Oncol. 2016;34(30):3664-3671. doi:10.1200/JCO.2016.67.4473

61. Wang A, Aragaki AK, Tang JY, et al. Statin use and all-cancer survival: prospective results from the Women's Health Initiative. Br J Cancer. 2016;115(1):129-135. doi:10.1038/bjc.2016.149

62. Escala-Garcia M, Morra A, Canisius S, et al. Breast cancer risk factors and their effects on survival: a Mendelian randomization study. BMC Med. 2020;18(1):327. 2020 Nov 17. doi:10.1186/s12916-020-01797-2

63. Amshoff Y, Maskarinec G, Shvetsov YB, et al. Type 2 diabetes and colorectal cancer survival: The multiethnic cohort. Int J Cancer. 2018;143(2):263-268. doi:10.1002/ijc.31311

64. Yuan C, Bao Y, Wu C, et al. Prediagnostic body mass index and pancreatic cancer survival. J Clin Oncol. 2013;31(33):4229-4234. doi:10.1200/JCO.2013.51.7532

65. Vornanen M, Konttinen H, Kääriäinen H, et al. Family history and perceived risk of diabetes, cardiovascular disease, cancer, and depression. Prev Med. 2016; 90:177-183. doi:10.1016/j.ypmed.2016.06.027
